# Soluble CD4 effectively prevents excessive TLR activation of resident macrophages in the onset of sepsis

**DOI:** 10.1038/s41392-023-01438-z

**Published:** 2023-06-19

**Authors:** Sheng-yuan Zhang, Qiu-ping Xu, Li-na Shi, Shih-wen Li, Wei-hong Wang, Qing-qing Wang, Liao-xun Lu, Hui Xiao, Jun-hong Wang, Feng-ying Li, Yin-ming Liang, Si-tang Gong, Hao-ran Peng, Zheng Zhang, Hong Tang

**Affiliations:** 1 CAS Key Laboratory of Molecular Virology and Immunology, Institut Pasteur of Shanghai, Chinese Academy of Sciences, Shanghai, 210031 China; 2 The Third People’s Hospital of Shenzhen, Shenzhen, 518112 China; 3 University of Chinese Academy of Sciences, Beijing, 100101 China; 4 The Laboratory of Genetic Regulators in The Immune System, Xin-xiang Medical University, Xin-xiang, Henan Province 453003 China; 5 The Joint Center of Translational Medicine, Guangzhou Women and Children’s Medical Center and Institut Pasteur of Shanghai, Guangzhou, 510623 China; 6 Department of Microbiology, Naval Medical University, Shanghai, 200433 China; 7 State Key Laboratory for Diagnosis and Treatment of Infectious Diseases, National Clinical Research Centre for Infectious Diseases, Collaborative Innovation Centre for Diagnosis and Treatment of Infectious Diseases, The First Affiliated Hospital, Zhejiang University School of Medicine, Hangzhou, 310003 China

**Keywords:** Inflammation, Innate immune cells

## Abstract

T lymphopenia, occurring in the early phase of sepsis in response to systemic inflammation, is commonly associated with morbidity and mortality of septic infections. We have previously shown that a sufficient number of T cells is required to constrain Toll-like receptors (TLRs) mediated hyperinflammation. However, the underlying mechanisms remains unsolved. Herein, we unveil that CD4^+^ T cells engage with MHC II of macrophages to downregulate TLR pro-inflammatory signaling. We show further that the direct contact between CD4 molecule of CD4^+^ T cells or the ectodomain of CD4 (soluble CD4, sCD4), and MHC II of resident macrophages is necessary and sufficient to prevent TLR4 overactivation in LPS and cecal ligation puncture (CLP) sepsis. sCD4 serum concentrations increase after the onset of LPS sepsis, suggesting its compensatory inhibitive effects on hyperinflammation. sCD4 engagement enables the cytoplasmic domain of MHC II to recruit and activate STING and SHP2, which inhibits IRAK1/Erk and TRAF6/NF-κB activation required for TLR4 inflammation. Furthermore, sCD4 subverts pro-inflammatory plasma membrane anchorage of TLR4 by disruption of MHC II-TLR4 raft domains that promotes MHC II endocytosis. Finally, sCD4/MHCII reversal signaling specifically interferes with TLR4 but not TNFR hyperinflammation, and independent of the inhibitive signaling of CD40 ligand of CD4^+^ cells on macrophages. Therefore, a sufficient amount of soluble CD4 protein can prevent excessive inflammatory activation of macrophages via alternation of MHC II-TLR signaling complex, that might benefit for a new paradigm of preventive treatment of sepsis.

## Introduction

Innate immune cells sense microbial pathogen-associated molecular patterns (PAMPs) via pattern recognition receptors (PRRs), which include Toll-like receptors (TLRs), NOD-like receptors (NLRs), C-type lectin receptors (CLRs) and nucleic acid sensors.^1–3^ The recognition upregulates both major histocompatibility complexes (MHC I and II) and co-stimulatory molecules, and secretes inflammatory cytokines, to prime subsequent adaptive immune response for protection.^4^

Hyperinflammation due to excessive activation of innate immune cells, however, leads to pathological alterations and correlates with the morbidity and mortality of infections.^5^ Innate inflammation therefore has to be tightly controlled by a series of negative regulators, at multiple levels, to maintain immunological homeostasis.^6,7^ We established previously that a sufficient amount of naïve T cells is required to dampen the acute innate inflammatory response to betacoronavirus and Gram-negative bacteria infections.^8,9^ To do so, T cells need to make direct cell-cell contact with antigen presenting cells (APC) to suppress their TLR-mediated inflammatory response, but independent of T cell receptor (TCR).^8^ Intriguingly, both effector and memory CD4^+^ T cells can block caspase-1/IL-1β activation by cell-cell contact with APC that diminishes NLRP3 and NLRC4 inflammasome activation.^10^

Sepsis is defined as an initial hyper-inflammatory response to systemic infection associated with a subsequent immune suppression and dysfunction, that can lead to multiple organ failure, secondary infections and mortality.^11^ The onset of sepsis is characterized by leukocytosis (marked increase in neutrophils and monocytes) in the first 2–4 days, followed by a state of lymphopenia as a result of apoptosis (drastic reduction of B cells, CD4^+^ and CD8^+^ T cells). Failure to restore cell numbers during either the stage of leukocytosis or lymphopenia results in increased mortality.^12,13^ Sepsis patients develope long-term immune impairment due to the reduction in the number and function of different immune cell populations.^14^ Patients who survive the early phase of sepsis develop immune suppression, which is characterized by T cell exhaustion, unresolved infection and susceptibility to opportunistic infection, and dysfunctional T cell repertoire.^13^ Compared to the understanding of functional impairment, however, the pro-apoptotic factors that cause T lymphopenia in sepsis and the impact of T lymphopenia to sepsis remain elusive.^15^ T lymphopenia, responding to systemic inflammatory response in the early phase of sepsis,^16^ is commonly associated with morbidity and mortality of septic infections.^17^ Such an acquired T-lymphopenia also occurs in certain autoimmune diseases and inflammageing process.^18^ Critically, insufficient number of naïve CD4^+^ T cells is responsible for the unleashed TLR inflammation with high morbidity and mortality rates.^3^ CD4 uses its extracellular domain to interact with β_2_-domain of MHC II,^19,20^ and MHC II is required for CD4^+^ T cells to suppress TLR hyperinflammation in APCs.^8,9^ Intriguing enough, MHC II is required to constrain B cell-response to LPS,^21,22^ and decreased expression of HLA-DR in monocytes and macrophages is observed in sepsis patients.^23^ Nevertheless, how CD4^+^ T cells signal through MHC II to suppress TLR inflammation in APCs remains unknown.

In this study, we demonstrate that CD4 molecule, either membrane bound or soluble ectodomain (sCD4), is necessary and sufficient to hamper TLR inflammation of APC and protect mice from the lethal septic inflammation. Furthermore, CD4 engagement recruits SHP2 and STING to the intracellular tail of MHC II in lipid rafts, which disrupts MHC II/TLR4 signaling complex, and inhibits TLR4 activation of IRAK1/TRAF6/NF-κB inflammatory response.

## Results

### CD4 T-lymphopenia accounted for the morbidity and mortality of sepsis

**Fig. 1:**
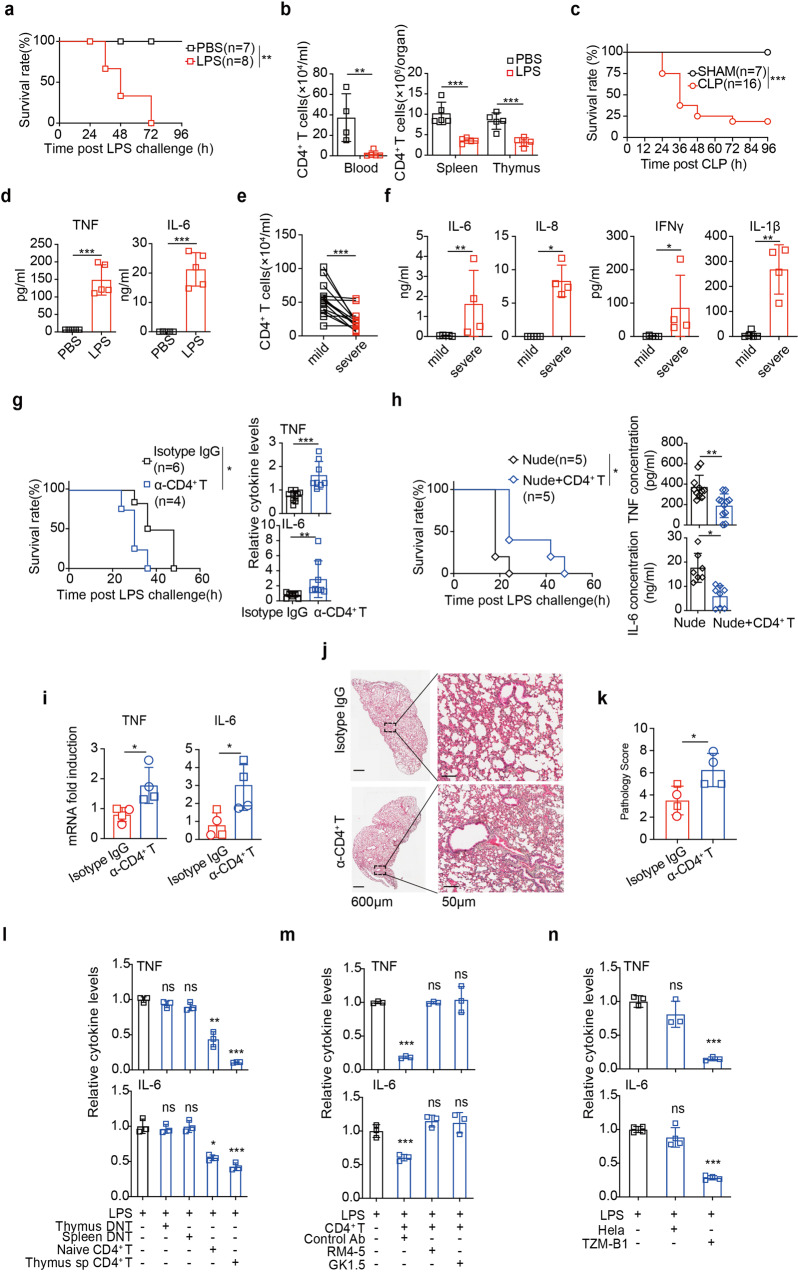
Membrane bound CD4 in T cells controlled TLR4 inflammation. **a** Survival rates, (**b**) CD4^+^ T cell counts in the indicated organs, and (**d**) serum TNF and IL-6 were measured 12 h after *i.p*. LPS (*n* = 7–8). **c** Survival rates were measured after CLP (*n* = 7–16). **e** Absolute numbers of peripheral CD4^+^ T cells, (**f**) indicated cytokines of BALF were measured in COVID-19 patients (*n* = 15). Survival rates and TNF/IL-6 levels were measured as in (**a**–**c**) except that CD4^+^ T cells were (**g**) pre-depleted (GK1.5) for 2 days, or (**h**) supplemented to nude mice for 7 days, before LPS injection. **i**–**k** hACE2 mice pre-treated with GK1.5 antibody or isotype IgG were infected with SARS-CoV-2 for 7days. **i** Fold changes of TNF and IL-6 mRNA normalized to *actin* in the lung by qPCR and (**j**–**k**) representative sections and pathology score of the lobe of left lung (with respective magnifications of areas of interest) on day 7. SARS-CoV-2 virus (*pfu* 1 × 10^2^ in box; *pfu* 1 × 10^5^ in circle). **l**, **m** Measurement of TNF and IL-6 in supernatants of BMDM 16 h after incubation with LPS (100 ng/mL), in the absence or presence of (**l**) T cells of the indicated origins (macrophages: T cells = 1:1), or (**m**) naive CD4^+^ T cells with the indicated blocking mAb against CD4 (RM4-5; GK1.5). **n** TNF and IL-6 in supernatants 3 h after LPS treatment of THP-1 cells co-cultured with HeLa or TZM-B1 cells. Mean ± SD are shown; *n* = 3–11 mice used where indicated; Statistics (ns, *P* > 0.05; *P < 0.05; **P < 0.01; ***P < 0.001): Log-rank (Mantel-Cox) test (**a**, **c**, **g** (left), **h** (left)), Unpaired *t* test (**b**, **d**–**f**, **g** (right), **h** (right), **i**, **k**), one-way ANOVA with Dunnett’s analysis (**l**, **m**, **n**)

We used LPS endotoxemia in mice as a surrogate of sepsis in patients^24^ to study the role of CD4^+^ T lymphopenia. C57BL/6 mice succumbed within 72 h post LPS *i.p*.injection (*hpi*, 10 mg/kg. Fig. 1a), accompanied with severe numerical reduction of both CD4^+^ (Fig. 1b) and CD8^+^ T cells (Supplementary Fig. 1a) in the thymus and periphery at 12 *hpi*, with the thymus drastically shrunk (Supplementary Figs. 1b, 2a–b). CD4^+^ T cells were more prone to sepsis-induced apoptosis, shown by reduced ratios of CD4/CD8 T cell counts in the thymus and spleen, as compared to the sham mice (Supplementary Fig. 1c). T lymphopenia was also observed in septic infection of mice by oral gavage of *Salmonella typhimurium* SL1344 (Supplementary Fig. 1d). To substantiate that LPS sepsis induced T lymphopenia can recapitulate that in the classical cecal ligation and puncture (CLP) sepsis (Fig. 1c), we compared side-by-side two models in the first 24 h after treatment. Both models showed qualitatively and quantativevly similar reduction of thymic sizes (Supplementary Fig. 2a) and increased pathology scores (Supplementary Table 4), as reflected by decreased thymocyte numbers, abnormal proportion of medulla and cortex and increased cell death (Supplementary Fig. 2b, c). Both models showed similar tendencies of T cell number reduction, decreased CD4/CD8 ratios except that in thymi of CLP model (Supplementary Fig. 2d,e). These results agreed with the previous CLP results.^15^ Therefore, T lymphopenia induced by a septic dose of LPS in mice manifested the dysregulated T cell response in CLP sepsis.

T lymphoneia associated with concomitantly elevated TNF and IL-6 in the serum at 12 *hpi* in LPS sepsis (Fig. 1d) or CLP sepsis (Supplementary Fig. 1e), among other pro-inflammatory cytokines and chemokines (PICC, Supplementary Fig. 1f). During the course of LPS sepsis (0–36 *hpi*), most PICCs reached the first and second peak at 6 and 16 *hpi*, respectively, with IL-6, IL-18, CCL5, CCL7 and CXCL1 sustaining at plateau at 36 *hpi* (Supplementary Fig. 1g). CD4^+^ T lymphopenia in circulation is also a major comorbidity of severe/critical COVID-19 patients who suffer from sepsis.^25,26^ We also observed that the inverse correlation of CD4^+^ (Fig. 1e) and CD8^+^ T cell counts (Supplementary Fig. 1h) and PICC levels (IL-6, IL-1β, IL-8 and IFNγ) in bronchoalveolar lavage fluids of COVID-19 patients hospitalized in 2020 (Supplementary Table 1), whose symptoms transformed from severe to mild pneumonia after symptomatic treatments (Fig. 1f).

Numerical and functional dysregulation of CD4^+^ and CD8^+^ T cells plays important roles in sepsis onset, progression and recovery.^12,13^ Insufficient number of CD4^+^ T cells per se led to the hyperinflammation and mortality in sepsis, because mice lacking CD4^+^ T cells, either by antibody-ablated (Fig. 1g) or in nude mice (Fig. 1h), were more susceptible to LPS sepsis (poorer survival and higher TNF/IL-6), compared to those by isotype antibody treatment (Supplementary Fig. 1i for antibody depletion efficiency) or CD4^+^ T cells adoptive transfer (Supplementary Fig. 1j for adoptive transfer efficiency), respectively. C57BL/6 mice of CD4^+^ T cells pre-depleted also showed augmented TNF/IL-6 response to SL1344 oral infection (Supplementary Fig. 1k) and much higher bacterial load (Supplementary Fig. 1l). Likewise, antibody depletion of CD4^+^ T cells in K18-hACE2 mice caused higher TNF/IL-6 expression (Fig. 1i) and more severe lung pathology (Fig. 1j, k) 7 d post SARS-CoV-2 infection. Therefore, aside from the functional exhaustion of T cells in the later stage of sepsis, the drastic reduction of CD4^+^ T cell numbers would attribute to the first hit of systemic hyperinflammation of sepsis.

To test critically whether CD4 molecule per se directly mediates CD4^+^ T-cell suppression of LPS/TLR4 inflammation, isolated T cells were co-cultured with bone marrow derived macrophages (BMDM) 2 h before LPS stimulation. T cells lacking CD4, either foreign antigen naïve (thymus DNT) or experienced (splenic DNT), failed to inhibit LPS activated TNF/IL-6 expression, except for naive or thymic single-positive CD4^+^ T cells (Fig. 1l). Therefore, CD4 moiety of naïve CD4^+^ T cells, but not TCR or TCR restriction as indicated previously,^8^ played an essential role to suppress TLR4 inflammation. This notion was substantiated by the co-culture experiments that anti-CD4 antibodies (RM4-5 or GK1.5) completely prevented CD4^+^ T cells from inhibiting TLR4 inflammation in macrophages (Fig. 1m). Of note, antibodies did not induce CD4^+^ T cell apoptosis (Supplementary Fig. 1m). To exclude the involvement of other CD4^+^ T-cell components, e.g., co-stimulatory receptors, we co-cultured THP-1 cells with TZM-b1 cells, a HeLa derivative that stably expresses human CD4.^27^ Only TZM-b1, but not HeLa cells, prevented THP-1 cells from TNF and IL-6 response to LPS (Fig. 1n). Collectively, CD4 molecule was necessary and sufficient to restrain TLR4 inflammatory response.

**Fig. 2:**
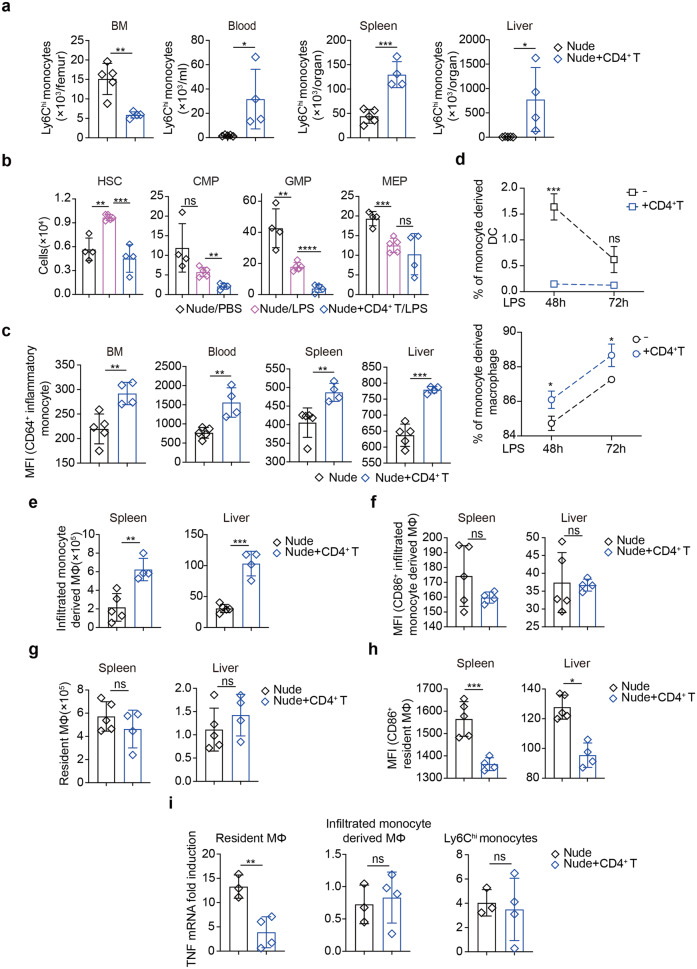
CD4^+^ T promoted monocytes differentiation and dampened resident macrophage hyperinflammation. **a**–**c**, **e**–**i** Nude mice or nude mice reconstituted with CD4^+^ T cells were treated *i.p*. LPS for 3 h before indicated analysis. Cell numbers of (**a**) Ly6C^hi^ monocytes, (**b**) progenitor cells in BM (HSC, GMP, CMP, MEP), and (**c**) activated monocytes (CD64^+^) in the indicated organs were flow cytometric analyzed. **d** Proportions of indicated cell types after in vitro differentiation of BM-derived monocytes co-cultured with CD4^+^ T cells, for the indicated time of LPS treatment. Cell numbers of (**e**) infiltrated monocyte derived macrophages or (**g**) resident macrophages and (**f**, **h**) their activation status (CD86^+^). **i** TNF mRNA induction by LPS in the indicated cell types sorted from the spleen. Mean ± SD are shown; *n* = 3–5 mice used where indicated; Statistics (ns, *P* > 0.05; *P < 0.05; **P < 0.01; ***P < 0.001): Unpaired *t* test

Acute LPS inflammation causes bone marrow (BM) monocytes to rapidly egress into circulation and infiltrate tissues, where they differentiate to inflammatory DCs and macrophages.^28^ Careful comparison of nude or nude mice adoptively transferred with CD4^+^ T cells (Supplementary Fig. 3a for the partial BM reconstitution) showed that, CD4^+^ T cell deficiency led to more Ly6C^hi^ monocytes retained in BM and less migrated to the blood or infiltrated to the spleen and liver 3 *hpi* (Fig. 2a, Supplementary Fig. 3b for gating strategy). This can be partially explained by the inhibitive effect of CD4^+^ T cells on TLR4 inflammation-driven emergency myelopoiesis in BM (reduced hematopoietic stem cells, common myeloid and granulocyte macrophage progenitors), with megakarayocyte and erythrocyte progenitors intact (Fig. 2b, Supplementary Fig. 3c for gating strategies of hematopoietic lineages). Lack of CD4^+^ T cells also suppressed monocytes activation (Ly6C^hi^ CD64^+^) in BM, the blood, and other indicated peripheral organs (Fig. 2c), likely due to insufficient IFNγ in the absence of CD4^+^ T cells (Supplementary Fig. 3e), as suggested previously.^29^ These results suggested that CD4^+^ T cells promoted emergency myelopoiesis and monocyte migration to inflamed tissues, necessary for inflammatory leukocytosis. In vitro co-culture experiments showed that CD4^+^ T cells accelerated TLR4-driven BM monocytes differentiation to macrophages, but DC significantly suppressed (Fig. 2d). Indeed, differentiation of spleen/liver-infiltrated monocytes to macrophages (CD11b^int^ F4/80^-^ MHC II^+^, Supplementary Fig. 3d for gating strategies) were increased by CD4^+^ T cells in endotoxemic mice (Fig. 2e), but not the activation of these monocyte-derived macrophages (CD86^+^, Fig. 2f). Surprisingly, the number of resident macrophages (CD11b^low^F4/80^hi^) in the spleen or liver remained unaffected by the presence or absence of CD4^+^ T cells (Fig. 2g), but their activation by LPS was effectively inhibited by CD4^+^ T cells (Fig. 2h). These results would suggest that resident macrophages played a more important role in mediating hyper-inflammatory response to CD4^+^ T lymphopenia in sepsis. qPCR measurement showed that TNF production was specifically reduced in splenic resident macrophages, but not infiltrated macrophages or monocytes, isolated 3 *hpi* (Fig. 2i). Therefore, CD4^+^ T cells would play profound roles in promotion of inflammatory leukocytosis and prevention of unleashed TLR4 inflammation of resident macrophages.

### sCD4 is sufficient to hamper innate inflammatory response

**Fig. 3:**
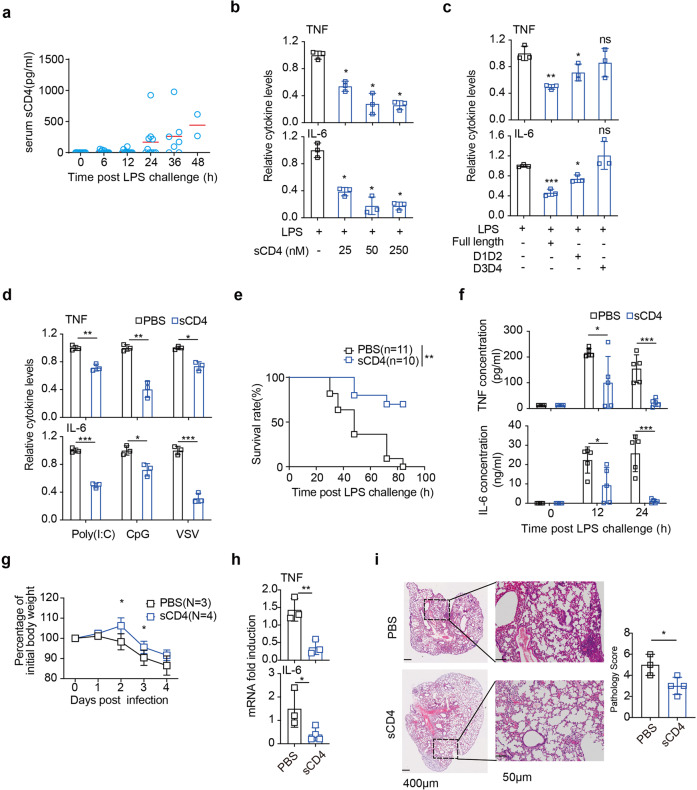
sCD4 protein effectively dampened TLRs inflammation. **a** ELISA measurement serum sCD4 after mice (*n* = 10) received LPS for the indicated time. TNF/IL-6 production 16 h after LPS stimulation of BMDM pre-incubated with 50 nM of (**b**) sCD4, (**c**) different ectodomains of sCD4, or (**d**) sCD4 but LPS was replaced with agonists for polyI:C (100 μg/mL), CpG-ODN (0.03 μM) or VSV (MOI = 5). **e** Survival rates and (**f**) serum TNF and IL-6 levels at the indicated time after WT mice were injected with sCD4 (10 mg/kg) (*n* = 10) or PBS (*n* = 11), 12 h before LPS challenge. **g**–**i** Changes of body weights after hACE2 mice pre-treated with sCD4 were infected with SARS-CoV-2 for 4 days (**g**). **h** TNF and IL-6 mRNA in lung by qPCR and (**i**) representative sections and pathology score of the lobe of left lung (with respective magnifications of areas of interest) on day 4. Mean ± SD are shown; *n* = 3–5 mice used where indicated; Statistics (ns, *p* > 0.05; *P < 0.05; **P < 0.01; ***P < 0.001): Log-rank (Mantel-Cox) test (**e**), Unpaired *t* test (**d**, **f**, **g**–**i**), one-way ANOVA with Dunnett’s analysis (**b**, **c**)

The membrane CD4 molecule (mCD4) is conventionally reckoned as a co-receptor of TCR signaling in T cells. Intriguingly, the ectodomain of CD4 molecule (sCD4, D1-D4 domains) circulates in the blood of infectious and autoimmune diseases.^30–32^ sCD4 in the serum steadily increased 24 h after mice received *i.p*. LPS (Fig. 3a), indicatve of a compensatory anti-inflammation function. Indeed, incubation of recombinant sCD4 with BMDM prevented TLR4 inflammatory activation (Fig. 3b), with D1-D2 domains being sufficient (Fig. 3c). sCD4 had no apoptotic effect on macrophages (Supplementary Fig. 4a). Naïve T cells dampen a wide range of TLR-mediated inflammatory response in various innate immune cells.^3^ sCD4 could also effectively prevent TLR3 (polyI:C), TLR7 (VSV), or TLR9 (CpG ODN) activation of macrophages (Fig. 3d), and inhibited the inflammatory response of bone marrow-derived dendritic cells (BMDC) to LPS, as well (Supplementary Fig. 4b). SARS-COV-2 Spike protein can directly target TLR4 to activate IL-1β expression.^33^ sCD4 protein effectively inhibited PICC response to SARS-CoV-2 infection of PBMC (Supplementary Fig. 4c). Critically, administration of sCD4 protein (200 μg/mice) to WT mice 12 h before LPS injection protected mice from the lethal challenge (Fig. 3e), with a significant reduction of TNF/IL-6 (Fig. 3f). sCD4 also prevented CLP-induced hyperinflammation similarly (Supplementary Fig. 4d), by effectively inhibiting the activation of splenic resident macrophages (Supplementary Fig. 4e). sCD4 did not affect the numerical distribution of CD4^+^ or CD8^+^ T cells in the thymus and peripherals of LPS (Supplementary Fig. 4f) or CLP (Supplementary Fig. 4g) model. Impressively, administration of sCD4 (300 μg/mice) 12 h before SARS-CoV-2 *i.n*. infection of K18-hACE2 mice attenuated the loss of body weight (Fig. 3g), reduced both PICC in the BALF (Fig. 3h) and pulmonary pathology (Fig. 3i). Therefore, CD4 molecule played a central role to control the homeostasis of TLR inflammatory responses of APCs, both in close contact (mCD4) and long range (sCD4).

### CD4/MHCII engagement suppressed macrophage inflammation

**Fig. 4:**
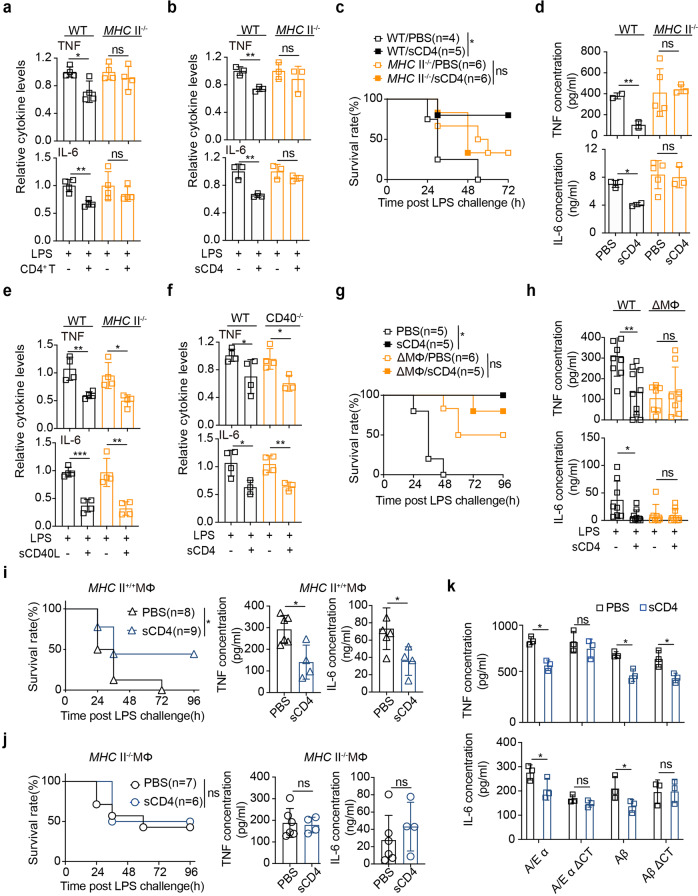
sCD4 downregulated TLR4 inflammation through MHC II in macrophages. **a**, **b** TNF/IL-6 measurement as in Fig. 1I except that MHC II^-/-^ BMDM were co-cultured with (**a**) an equal number of CD4^+^ T cells, or (**b**) 25 nM sCD4 protein. **c** Survival rates and (**d**) serum TNF/IL-6 24 h after LPS *i.p*. injection in wt littermates or MHC II^−/−^ mice pre-treated with a single dose of sCD4 (10 mg/kg). TNF/IL-6 in supernatants after (**e**) MHC II^−/−^ BMDM were pre-treated with 25 nM sCD40L, or (**f**) CD40^−/−^ BMDM cells with 25 nM sCD4 before LPS stimulation. Survival rates and serum TNF/IL-6 in mice with (**g**–**h**) macrophages ablated (ΔMΦ) or (**i**–**j**) ΔMΦ mice reconstituted with either MHCII^+/+^ or MHCII^−/−^ macrophages, that received 10 mg/kg sCD4 or PBS before *i.p*. LPS stimulation. **k** TNF/IL-6 in the supernatants 12 h after peritoneal macrophages that transiently overexpressed with the indicated MHCII subunits or cytoplasmic tail-truncational mutants (ΔCT) were stimulated with LPS or LPS plus 25 nM sCD4. Mean ± SD are shown; *n* = 3–10 mice used where indicated; Statistics (ns, *P* > 0.05; *P < 0.05; **P < 0.01; ***P < 0.001): Log-rank (Mantel-Cox) test (**c**, **g**, **i** (left), **j** (left)), Unpaired *t* test (**a**–**b**, **d**, **e**–**f**, **h**, **i** (right), **j** (right), **k**)

MHC II is required for T cells to hamper innate inflammation.^8^ Specifically, MHC II transmitted the inhibitive signal of CD4 to TLR4 activation, because neither isolated CD4^+^ T cells (Fig. 4a) nor sCD4 (Fig. 4b) were able to prevent LPS activation of MHC II-deficient (MHC II^−/−^) macrophages in vitro, or in MHC II^−/−^ mice (Fig. 4c, d). CD40L of CD4^+^ T cells can also downregulate macrophage TNF response to LPS through a paracrine activation of IL-10.^34^ This process did not require MHC II, because trimeric CD40L protein (sCD40L) still inhibited TNF/IL-6 response to LPS in MHC II^−/−^ macrophages in vitro (Fig. 4e). On the other hand, sCD4 inhibited LPS inflammation as efficient in CD40^-/-^ BMDM as in wt macrophages (Fig. 4f). Therefore, CD4 and CD40L of CD4^+^ T cells would function independently to prevent APC from TLR overactivation.

To critically test whether MHC II in macrophages per se is sufficient to mediate sCD4 antiphlogistic effect, macrophages were first depleted by clodronate liposome (Supplementary Fig. 5a). Macrophages depleted mice (ΔMΦ) partially resisted to lethal dose of LPS challenge (Fig. 4g), accompanied with reduced TNF/IL-6 (Fig. 4h). Consequently, sCD4 inhibited TLR4 inflammation only when MHC II^+/+^ (Fig. 4i), but not MHC II^─/─^ (Fig. 4j) macrophages were adoptively transferred to ΔMΦ mice. To further determine which subunit of MHC II was required to transmit the reverse signal of sCD4, different truncational mutants of MHC II subunits fused with eGFP were overexpressed in isolated peritoneal macrophages (Supplementary Fig. 5b for equal ectopic expression). Deletion of the cytoplasmic tails of MHCII A/E-α and A-β abrogated the inhibitive effect of sCD4 on TNF/IL-6 response (Fig. 4k). Therefore, these results suggested that MHC II in macrophages was sufficient to transmit CD4 reverse signal to suppress TLR hyperinflammation.

### SHP2 was required to mediate MHC II crosstalk to TLR pathway

**Fig. 5:**
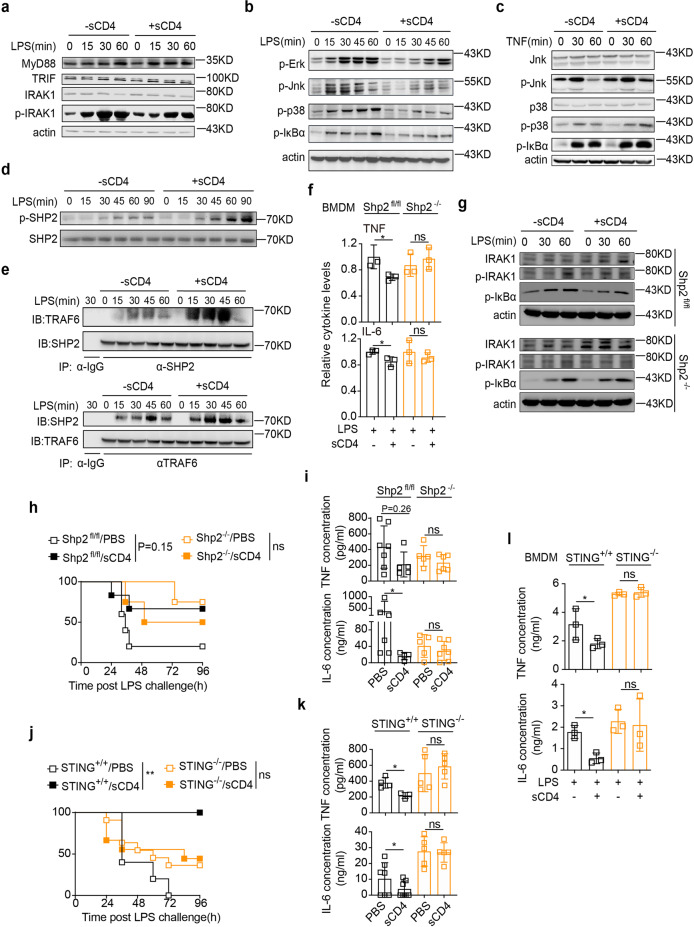
SHP2 and STING mediated CD4/MHC II crosstalk to TLR signaling. BMDM cells were incubated with (**a**–**b**, **d**–**e**) 100 ng/mL LPS or (**c**) 5 ng/mL TNF in the presence or absence of 25 nM sCD4 for the indicated time. **a**–**d** Western blotting of the indicated proteins. **e** Reciprocal co-immunoprecipitation between SHP2 and TRAF6 in pm cells. **f** TNF/IL-6 in supernatants were measured as in Fig. 3B except for that SHP2^−/−^ BMDM used. **g** Western blots as in panels (**a**–**b**) except that SHP2^−/−^ BMDM were used. SHP2^fl/fl^ macrophages were used as controls. **h** Survival rates and (**i**) serum TNF/IL-6 were measured at the indicated time after *i.p*. LPS in macrophage specific SHP2^−/−^ mice that received sCD4 (10 mg/kg). **j** Survival rates and (**k**) serum TNF/IL-6 levels 12 h post LPS injection of STING^−/−^ mice. **l** TNF/IL-6 in supernatants 4 h after LPS treatment of BMDM isolated from STING^−/−^ or wt mice in the absence or presence of sCD4 (25 nM). Mean ± SD are shown; *n* = 3–6 mice used where indicated; Statistics (ns, *P* > 0.05; *P < 0.05): Unpaired *t* test (**f**, **i**, **k**, **l**), Log-rank (Mantel-Cox) test (**h**, **j**)

LPS is sensed by TLR4/MyD88, which recruits and phosphorylates IRAK1 to activate MAPK and NF-κB signaling.^35^ The previous results show that the cytoplasmic tail of MHC II can contact MyD88 to inhibit TLR4 signaling.^36^ We then set to test whether sCD4 engagement of MHC II would take advantage of the same pathway to crosstalk to TLR pathway. Phosphorylation of IRAK1 in BMDM (Fig. 5a), as well as Erk, Jnk, p38 and IκBα (Fig. 5b and Supplementary Fig. 6a), in response to LPS was effectively inhibited by sCD4. Importantly, sCD4/MHC II ligation antagonized specifically the TLR signaling, because TNFR mediated MAPK or NF-κB activation was not affected (Fig. 5c). There are few reports that IRAK1 phosphorylation can trigger its proteosomal degradation, that may help reducing TLR4/NF-κB activation.^37,38^ The total IRAK1 protein level slightly decreased even after a longer time of LPS stimulation (Fig. 5a, g). More experiments are needed to determine the role of IRAK1 degradation in MHCII reverse signaling.

Tyrosine phosphorylation plays an important role in the negative regulation of TLR signals.^39^ The global tyrosine phosphorylation in LPS-treated BMDM was increased by sCD4 (Supplementary Fig. 6b), where SHP2 phosphorylation (Fig. 5d), but not Btk, Syk or SHP1 (Supplementary Fig. 6c), was specifically enhanced. SHP2 is a protein tyrosine phosphatase that regulates MyD88 signaling by contact with TRAF6.^40^ LPS-induced binary interaction between SHP2 and TRAF6 was augmented by sCD4, as measured by reciprocal co-immunoprecipitation assays (Fig. 5e). Therefore, SHP2 would mediate CD4/MHC II crosstalking to TLR/MyD88 pro-inflammatory signaling. Indeed, sCD4 failed to inhibit TNF/IL-6 response to LPS in SHP2-deficient BMDM (Fig. 5f), where IκBα (NF-κB) and IRAK1 (MAPK) activation was restored (Fig. 5g). In mice with SHP2 specifically ablated in macrophages, sCD4 no long improved the survival rates of LPS sepsis (Fig. 5h), nor inhibited TNF/IL-6 response (Fig. 5i). Besides MyD88,^36^ STING may associate with the membrane proximal/cytoplasmic domain of MHC II, probably through CD79, in APC cells.^41^ STING was also required for CD4/MHC II reverse signaling, since sCD4 no long improved the survival rates of LPS sepsis (Fig. 5j), nor inhibited TLR4 inflammation in eithr STING^−/−^ mice (Fig. 5k) or STING^−/−^ macrophages in vitro (Fig. 5l). Together, these results suggested that SHP2 and STING might bridge CD4/MHC II inhibitive crosstalk to TLR4/MyD88 pro-inflammatory signaling.

### sCD4 engagement dissociated the membrane raft domain of MHC II and TLR4

**Fig. 6:**
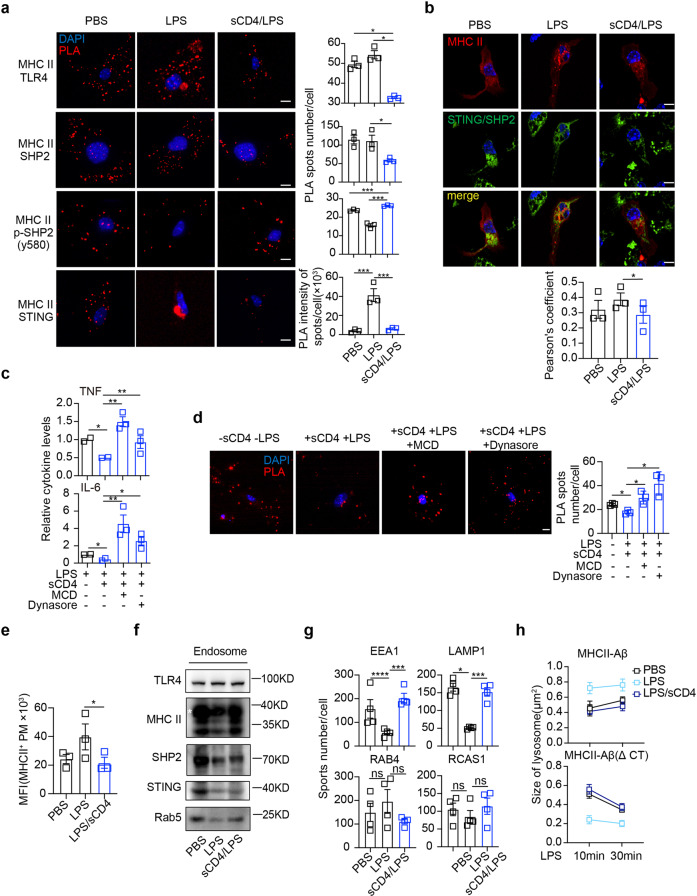
sCD4 disrupted MHCII/TLR4 rafts and reduced LPS/TLR4 inflammatory membrane confinement. Duolink assays to quantify protein-protein interactions of (**a**) the indicated pairs (red), or (**b**) between STING and SHP2 (green) that combined with immunofluorescent staining of MHC II (red) in BMDM. Tripartite colocalization indicated in yellow. The nuclei counter-stained with DAPI. Pearson’s coefficients indicated the degree of colocalization. Bar = 5 μm. **c** TNF and IL-6 in supernatants 30 min after peritoneal macrophages treated with LPS or LPS plus sCD4, in the presence of indicated endocytosis inhibitors. The average of two independent repeats. Three replicate wells were used for each condition where the indicated inhibitor was added. **d** Duolink spots of MHC II-SHP2 interactions and (**e**) flow cytometric analyses of cell surface MHC II. Bar = 5 μm. Macrophages (5 x 10^6^) were treated with LPS/sCD4 for 1 h, and (**f**) endosomes were isolated for immunoblot analysis of the indicated proteins (asterisk), or (**g**) organelle numbers per cell were quantified after immunofluorescence staining of EEA1 (early endosomes), LAMP1 (lysosomes), RAB4 (recycling endosome) and RCAS1 (Golgi). **h** Lysosomes size was quantified using LysoTracker after RAW264.7 cells were transfected with GFP-tagged MHC II Aβ or MHC II AβΔCT. Several view fields were randomly selected and images were acquired every 10 s for 20 min of LPS or LPS plus sCD4 treatment. Mean ± SD are shown; *n* = 3–4 mice used where indicated; Statistics (ns, *P* > 0.05; *P < 0.05; **P < 0.01; ***P < 0.001;): Unpaired *t* test

Because the cytoplasmic tail of MHC II is short and devoid of ITIM or ITAM motif, it was critical to test whether and how STING and/or SHP2 directly tethered to MHC II. Duolink proximity ligation assays (PLA) were performed to measure these potential intermolecular interactions (Fig. 6a, red dots). At the resting state of cultured BMDM, SHP2 and STING, including activated SHP2 (pY580), were associated with MHC II. The interactions between MHC II and TLR4 in the cytoplasmic membrane, and MHC II with STING oligomerized in ER/Golgi^42^ activated by LPS, were effectively disrupted by sCD4 (Fig. 6a). LPS dissociated the activated SHP-2 with MHC II, which could be effectively reverted by sCD4 (Fig. 6a). Merge of fluorescent antibody labeled MHC II (red) with Duolink dots of SHP2/STING (green) showed that SHP-2 and STING could form complex and were in close proximity to MHC II, and sCD4 reduced the assembly of MHCII-SHP2/STING complex (Fig. 6b). Furthermore, sCD4-increased pSHP-2/MHC II interaction was abolished in TLR4^−/−^ BMDM (Supplementary Fig. 7a), suggesting that SHP-2, probably through complexing with STING, function to couple MHCII-TLR4 crosstalk for sCD4. These results would suggest that sCD4 dissociated LPS/TLR4 from MHC II/STING complex with presumably assistance of the activated SHP2.

LPS confines TLR4 in membrane rafts so as to transmit pro-inflammatory signaling.^30,43^ Inhibitors of lipid raft- (MCD) or clathrin- (Dynasore) mediated endocytosis impaired the ability of sCD4 to antagonize TLR4 inflammation (Fig. 6c) or dissociate SHP2 from MHC II (Fig. 6d). Therefore, the pro-inflammatiory LPS/TLR4 raft compartments might be disrupted by sCD4 engagement of MHCII membrane domains. On the other hand, sCD4 abrogated MHC II upregulation in BMDM surface by LPS, both by fluorescence staining (Fig. 6e and Supplementary Fig. 7b) and and flow cytometry detection (Supplementary Fig. 7c), suggesting that sCD4 engagement might augment MHC II internalization^44–46^ and/or lysosomal targeting.^46^ LPS reduced overall the formation of early endosomes (Rab5), and concomitant endosomal distribution of MHC II, SHP2 and STING. sCD4 treatment, however, significantly restored their endosome trafficking (Fig. 6f). In addition to increased early endosome formation (EEA1), sCD4 also accelerated LAMP1-stained lysosome formation (Fig. 6g and Supplementary Fig. 7d) of MHC II complex. Live cell imaging indicated that sCD4-driven endosome-lysosome fusion relied on the cytoplasmic domain of MHC II-Aβ (Fig. 6h), in agreement with the previous finding that MHC II cytoplasmic domain controls its endocytotic presentation.^47^ Therefore, these results suggested that sCD4 disrupted pro-inflammation raft domains of MHC II/TLR4 complex, and by augmenting MHC II endocytosis, to destabilize pro-inflammatory membrane confinement of TLR4.

## Discussion

T lymphopenia occurs in the early phase of septic hyperinflammation,^16^ is commonly associated with morbidity and mortality of septic infections.^17^ Whether and how such a stoichiometric control of innate hyperinflammation is rooted in numerical reduction of naive CD4^+^ T cells, or CD4 molecule per se, remains elusive. This work establishes unambiguously that systemic hyperinflammtion upon infection was aggrevated by the loss of CD4^+^ T cells, that unleashed the restrains of MHC II/TLR4 inflammatory "signalosome" for cytokine storm or cytokine release syndrome in sepsis. This conclusion does not intend to exclude the contribution of CD8^+^ T lymphopenia in sepsis also observed in LPS and CLP models. Previous report showed that CD8^+^ T cells are able to engage MHC I to inhibit TLR inflammation.^40^

CD4 molecule has been extensively studied, both structurally and functionally, as a co-receptor of TCR in facilitating MHC II-restricted T-cell recognition.^48^ CD4 contacts a conserved membrane-proximal region of MHC II, an action independent of TCR.^49^ It has long been established that CD4/TCR complex uses MHC II as a receptor to feedforward activate antigen presentation signaling.^50^ However, whether and how CD4/MHC II engagement negatively regulates APC inflammatory response to invading pathogens has never been addressed before. We revealed in this work that, CD4 molecule may function as an inhibitory ligand of MHC II, independent of TCR or CD40L, to constrain TLR inflammatory activation of resident macrophages, rather than monocyte-derived macrophages that had infiltrated to the lesion. CD4/MHC II engagement to downregulate TLR inflammation did not involve APC apoptosis, apparently different from the actions of antibodies or cognate TCR engagement of MHC II that accelerate cell death of activated DC,^51,52^ macrophages^53,54^ and B cells,^55,56^ in caspase-independent, PKC/ERK-dependent manner. This and the previous work^8,57^ also showed that TCR engagement is not essential for naive CD4^+^ T cells to inhibit PICC response in APC. Of course, CD4^+^ T cells may utilize co-inhibitory molecules, BTLA^58^ or CD40L,^34^ to down-regulate TNF response to LPS. CD40L signaling in effector/memory CD4^+^ T cells, independent of TCR, inhibits IL-1β secretion by APCs.^10^ Other TNF superfamily members, RANKL, LIGHT, 41BBL, CD30L and OX40L, may also be involved in such an inhibitory T-APC immunological synapse. This work further show that CD4 molecule functions independent of CD40L, and not involving TNFR signaling, that essentially regulates the homeostatic APC inflammatory response.

Previous studies using antibody cross-linking,^59^ super antigens^60,61^ or lymphocyte-activating gene-3 (LAG-3), a MHC II ligand,^62^ demonstrate that the heterodimeric MHC II protein complex can function as a receptor to activate T-dependent B cell response. In this context, an array of trans-membrane proteins, including CD79, CD19, CD20, CD21, CD40 and among others, physically associate with MHC II for B cell activation and differentiation.^50^ Intriguing enough, we showed additionally that SHP2 activation and association with MHC II cytoplasmic tail, but not SHP1, was required for CD4 inhibition of TLR4/MyD88 signaling and IRAK1/Erk activation. STING/SHP2 interaction has been shown in cytosolic DNA activated JAK1/STAT1 signaling.^63^ This is in line with previous observations that STING promotes MHC II-aggregation induced B lymphoma cell death and Erk activation independent of SHP1,^64^ albeit SHP1 can associate with STING.^65^ SHP2 also inhibits TLR3/TRIF signaling pathway in human monocytes.^66^ It remains to test whether CD4 inhibition of TLR3 inflammation requires SHP2. Furthermore, sCD4/MHC II engagement did not alter LPS-induced phosphorylation of Btk, different from intracellular MHC II, which can interact and activate Btk via CD40 to promote endosomal TLR signaling.^67^ Therefore, it is tempting to speculate that cell surface and intracellular MHC II may use different mediators to sort TLR signaling.

Importantly, we showed that increased serum sCD4 associated with LPS hyperinflammation. sCD4, presumably shed by a MMP-like sheddase, is also increased in patients of chronic inflammatory diseases, and correlates positively with the disease activities and poor prognosis in RA patients.^68^ This would emphasize a compensatory effect of the increased sCD4 in control of inflammation, albeit sCD4 at sub-nanomolar concentrations in these patients is insufficient to do so. The binding affinity of CD4 ectodomain to pMHC II (~150–200 μM) is relatively weak as measured in vitro.^69^ This far exceeds the concentrations of sCD4 needed for 50% inhibition of TLR4 hyperinflammation in vitro (~25 nM) or in vivo (~125 nM). We reason that auxiliary factors assembled within MHC II/TLR compartments in macrophages might help offset the low affinity of CD4 to MHC II. The exact makeup and function of MHC II/TLR rafts targeted by CD4 in this context remain to be determined. At least, TNFR is not associated with MHC II/TLR ‘signalosome’. A nanomolar IC_50_ would potentially make sCD4 (~10–15 mg/kg) especially desirable to prevent the onset of fatal sepsis. This work thus indicated that sCD4 might provide a new antiphlogistic paradigm to prevent severe/critical SIRS or sepsis. Applying sCD4 protein to target inflamed innate cells is apparently advantageous over the current therapeutics,^70^ e.g., corticosteroid or antibodies, that target PICC or their cognate receptors (Supplementary Fig. 9).

## Materials and methods

### Mice

Mice homozygous null for MHC II genes (B6.129S-H2^dlAb1-Ea^/J) were kindly provided by Dr. Xue-tao Cao (National Key Laboratory of Medical Immunology, Naval University of Medicine, Shanghai, China), SHP-2^fl/fl^ and LysM-cre knock-in mice by Drs. Hui Xiao and Gen-Sheng Feng, STING^−/−^ mice by Dr. Xin-wen Chen (Wuhan Institute of Virology, Chinese Academy of Sciences). K18-hACE mice (C57BL/6 background) were purchased from Gem Pharmatech (Jiangsu, China). CD40-deficient mice were generated by CRISPR-Cas9 approach. Guide RNA sequences were listed in Supplementary Table 2. Littermates of 6–8 weeks (body weight and gender matched) were used, unless otherwise mentioned. Where used, nude mice, BABL/c and C57BL/6J mice were from Vital River Lab Animal Tech (Beijing). All mice were bred under specific pathogen-free conditions, founder mice and breeding littermates were genotyped by PCR (primers in Supplementary Table 3, and results in Supplementary Fig. 8). All animal experiments were performed in accordance with institutional guidelines and were approved by the Animal Care and Use Committees of the Institute of Biophysics or Institut Pasteur of Shanghai, Chinese Academy of Sciences (No. A2020043).

### Reagents and antibodies

LPS (0111:B4), CpG ODN and poly (I:C) were purchased from Sigma-Aldrich (St Louis, USA). Mouse recombinant CD4 (His-tag), CD40L (Fc-tag) and TNF (Fc-tag) proteins were from Sino Biological. D1-D2 (Fc- or 6xHis-tag) and D3-D4 (His-tag) of CD4 were generated by Bac-to-Bac Baculovirus Expression System (FulenGen). LysoTracker Deep Red was from Life Technologies. Antibodies against indicated proteins used in this study were: CD4 (GK1.5 and RM4-5) from eBioscience, mouse IgG2a isotype control antibody (401501) and MHC class II (107610) from BioLegend; TRIF (ab13810), β-actin, Goat anti-Rat Alexa Fluor 647(A-21247), Donkey anti-Mouse IgG Alexa Fluor 488(A-21202) and Goat anti-Rabbit Alexa Fluor 555(A-21429) from Thermo Fisher; TRAF6 (sc-8409) from Santa Cruz; MyD88 (D80F5), IRF3 (D83B9), Syk (D3Z1E), Btk (D3H5), IRAK1 (D51G7), Erk (9102), IgG (7074, 7076), Jnk (56G8), p38 (9212), SHP-2 (D50F2), phospho-Erk at Thr202-Tyr204 (E10), phospho-Jnk at Thr183-Tyr185 (G9), phospho-SHP-2 at Tyr580 (5431), phospho-p38 at Thr180-Tyr182 (9211), phospho-IRF3 at Ser396 (4D4G), phospho-IκBα at Ser32-Ser36 (5A5), phospho-Btk at Tyr223(D9T6H), phospho-Syk at (Tyr352), phospho-Tyrosine antibody (p-Tyr-100), EEAL (C45B10), LAPM1 (D2D11), RAB4 (2167T) and RCAS1(D2B6N) from Cell Signaling Technology; RAB5(AR038) from Beyotime. SHP1 (ab32559), SHP-2 (ab131541), phospho-SHP1 at Y536 (ab51171), anti-MHCII (ab180779), anti-MHCII (ab25681), anti-TLR4 (ab22048), anti-SHP2 (ab131541), anti-P-SHP2 (ab62322) mouse IgG isotype control (ab172730), rabbit IgG isotype control (ab37355) from Abcam; STING (66680-1-lg) from Proteintech. Phospho-IRAK1 at T209 (ab218130) from Sigma-Aldrich.

### Mouse models of sepsis

The endotoxemia mouse model was established by *i.p*. injection of LPS (8–10 mg/kg body weight) in either C57BL/6J or nude mice. Where indicated, mice were *i.p*. injected with sCD4 (10 mg/kg) 12 h before LPS *i.p*. injection or transferred with CD4^+^ T cells 7 days before LPS injection. The cecal ligation and puncture (CLP) induced polymicrobial sepsis were performed as previously described.^71^ Briefly, the mice were anesthetized, and a small midline abdominal incision was made. The cecum was then exteriorized, and the distal three-quarters of the cecum was immediately ligated without causing intestinal obstruction. The ligated cecum was punctured with an 18-gauge needle, and a small amount of feces was gently squeezed out of the perforation to ensure patency of the punctures. The cecum was then relocated into the abdominal cavity, and the incision was closed. In the sham surgical controls, the cecum was exposed without ligation or puncture. At the indicated time points, mice were euthanized and the intestine, thymus, liver, spleen, and peripheral blood were collected for analysis. Thymus were fixed in 4% PFA solution and for histological stainings.

### Histology

Left lobes of lung or thymus were fixed in 4% paraformaldehyde and embedded in paraffin. Fixed tissues were sliced into 5 μm thick sections and stained with hematoxylin and eosin (H&E). To obtain a whole slide image, the slides with Mount coverslip onto the section on glass slide with neutral resins and subsequently the imaging data were obtained with Vectra3 with 20× objective (PerkinElmer, Vectra 3). Degrees of pathological alteration were scored following the standards described in Supplementary Table 4.

### CD4^+^ T cell depletion and adoptive transfer

CD4^+^ T cell depletion was performed as previously described using CD4 antibody (200 μg in 250 μL GK1.5, *i.p*.) or isotype antibody every 3 days. Splenic CD4^+^ T cells were enriched with mouse CD4^+^ T cell negative isolation kit (Easy Sep^TM^, >95% purity, Stemcell, Canada) and *i.p*. transferred to recipient mice (2 × 10^6^ cells/mouse) for 7 days.

### Salmonella typhimurium infection

*Salmonella typhimurium* (strain SL1344) was grown overnight at 37 ^o^C and then subcultured the next day in fresh LB to OD_600_ = 0.6–0.8 at 37 ^o^C. Water and food were withdrawn 4 h before gavage, mice (C57/B6j, male, 8 weeks old) were inoculated with 1 × 10^8^ bacteria by oral gavage. Drinking water and food were supplied immediately. After 60 h, mice were euthanized and the blood, and spleen were harvested for bacterial loads, cytokines and immune cells analyses.

### COVID-19 patients

#### Study approval

This study was approved by the Research Ethics Committee of Shenzhen Third People’s Hospital, China (approval number: 2020-181) and the written informed consents were obtained from enrolled patients.

#### Patients and samples

The study enrolled 15 patients (male:female = 13:2; ages 36 to 73) diagnosed positive for SARS-CoV-2 infection in January 2020 (Supplementary Table 1, qPCR, nasopharyngeal swab and throat swab specimens), and negative for influenza A/B, RSV or adenovirus co-infection. Chest computed tomographic scans showed varying degrees of bilateral lung patchy shadows or opacity. Their clinical manifestations were determined as according to the Diagnosis and Treatment Protocol of COVID-19 (the 7th Tentative Version) by National Health Commission of China issued on March 3, 2020. Severe patients were diagnosed based on one of the following criteria: (1) Respiratory distress: RR ≥ 30 times/min; (2) Fingertip oxygen saturation ≤93% at resting state; (3) Arterial partial pressure of oxygen (PaO2)/fraction of inspiration oxygen (FiO2), P/F ≤300 mmHg (1 mmHg = 0.133 kPa); (4) Patients with obvious progress of lesions in 24–48 h shown by pulmonary imaging >50%.

All patients were hospitalized at Shenzhen Third People’s Hospital 1 to 16 d after symptom onset. Among these patients, 11 had exposure history after traveling to Wuhan and other 4 had direct contact with COVID-19 patients from Wuhan. All patients presented with fever, fatigue and dry cough, and 5 had developed severe pneumonia when admitted to the hospital (patient number C3, C4, C8, C11, C14). Eleven patients had underlying disease such as hypertension, cardiopathy, autoimmune disorder or chronic hepatitis B. All patients progressed from mild to severe pneumonia, or severe to mild pneumonia, and all recovered and discharged after symptomatic treatment. Patient C11 succumbed to COVID-19 in hospital. All patients received interferon and ribavirin and lopinavir and/or methylprednisolone treatments.

#### Isolation of BALF and PBMC

BALF samples were obtained and placed on ice, passed through a 100 µm nylon cell strainer to remove clumps and debris, centrifuged (1000 × *g*, 10 min). Supernatants (25 µL) were mixed with equal volume of sonication beads and detection antibodies for IL-1β, IL-2, IL-4, IL-5, IL-6, IL-8, IL-10, IL-12p70, IL-17, IFN-α, IFN-γ or TNF-α (Uni-medica, Shenzhen, China) on a shaker (500 rpm, room temperature, 2 h). SA-PE (25 µL) was added directly to each tube and mixed for 30 min before flow cytometric analysis (Canto II, BD). Data were analyzed using LEGENDplex v8.0 software (VigeneTech Inc.). Anticoagulant blood was used to measure CD3^+^CD4^+^ and CD3^+^CD8^+^ T cell counts by flow cytometry. All procedures were performed within 2 h after specimen collection in a BSL-3 laboratory approved by Ethics Committees of Shenzhen Third People’s Hospital (SYB3-2020003). PBMC was isolated from blood samples using standardized density gradient technique (Ficoll-Paque).

### SARS-CoV-2 infection of PBMC

PBMC (5 × 10^5^ cells/ml) were seeded in 96-well plate with RPMI-1640 for 12 h before incubation with 50 nM sCD4 for additional 2 h. Cells were then infected with SARS-CoV2 (separated from patient YCJ. MOI = 0.1 or 0.5, respectively). Supernatants were collected after 24 h of infection, and the virus was inactivated using β-lactone (Solarbio) at 4 ^o^C for 36 h. TNF and IL-6 were measured by by using Elisa kit (BioLegend). Experiment perfomed in a Bio-safety Level 3 facility.

### SARS-CoV-2 infection of hACE2 transgenic mice

Mice were used following institutional ethics guidelines and approved by the Animal Care Committee of Naval Medical University (No.# NMUP3LP20201109-2). K18-hACE2 transgenic C57BL/6 mice (male, 10 weeks old) were *i.p*. injected 500 μg of GK1.5 antibody (in 200 µL PBS) to deplete CD4^+^ T cells 24 h before infection. For sCD4 treatment, 300 µg of sCD4 (in 200 µL PBS) was *i.p*. administered 12 h before infection. SARS-CoV-2 virus (pfu 1 × 10^2^ in a final volume of 50 μL PBS) was *i.n*. infected. Mice were then euthanized at 7 d post infection (CD4^+^ T cell depeletion group) or 4 d (sCD4 pre-treatment group), and lungs were harvested for indicated analyses.

### Plasmid construction and transfection

MHC II A/Eα (NM_010378.3) and Aβ (NM_207105.3) cDNA were subcloned into vector pEGFP-N1. The cytoplasmic domain of MHC II was replaced with 3 x Gly (designated as MHCII A/Eα ΔCT and AβΔCT). For transient expression of the indicated MHC II chains, the plasmids were electroporated to peritoneal macrophages using the Neon Transfection System (ThermoFisher). Typically, 1 × 10^6^ cells were transfected with 1 μg plasmid (1200v, 30 ms; twice).

### Flow cytometry

DN T cells, monocytes and macrophages in the spleen, or DN T and SP CD4^+^ T cells in the thymus were isolated (1 × 10^7^ splenocytes/thymocytes labeled with 5 μg indicated antibodies in 1 mL staining buffer (PBS, 1% FBS)) by FACSAria II sorter (BD Biosciences). Multicolor flow cytometric analyses were routinely performed on LSR Fortessa (BD Bioscience) and analyzed by FlowJO software (Tree Star, OR). Dead cells were excluded with 7-AAD (eBioscience) or DAPI (BD Biosciences). Detailed antibodies information in Supplementary Table 5. Analysis of apoptosis was performed with Annexin V-FITC Apoptosis Detection Kit according to the manufacturer’s instruction (BD Biosciences).

### Co-culture and PICC measurements

Bone marrow cells were isolated from 6–8 week old C57BL/6 mice. After red blood cells were lysed with RBC lysis buffer (eBioscience), cells were seeded in RPMI-1640 medium containing 10% FBS (Gibco) and 1% Pen-Strep. M-CSF (20 ng/mL, PeproTech), or GM-CSF (20 ng/mL) and IL-4 (10 ng/mL, PeproTech), were used for BMDM or BMDC differentiation, respectively. BMDM and BMDC were used on days 7–8, with fresh media containing cytokines replaced every 2 days. CD4^+^ T cells (5 × 10^4^) were co-cultured 1:1 with differentiated BMDMs or BMDCs for 2 h. Where indicated, 25 nM sCD4 or sCD40L protein (Sino Biological) was added to the culture overnight. BMDM or BMDC cells were seeded in a 96-well plate and stimulated with 100 ng/mL LPS, 100 μg/mL poly(I:C), 0.3 μM CpG ODN or VSV (MOI = 5) for 16 h, before the supernatants were collected for cytokine measurements by Luminex analyses using the Procartaplex Mouse 2-plex kit (eBioscience), Mouse 26-plex kit (Thermo Fisher) or Human 11-plex kit (eBioscience). The concentration of cytokines were detected and analyzed by Bio-Plex 200 System (Bio-Rad).

### ELISA analysis of serum sCD4

To measure mourse CD4 in sera, 96-well high binding plates (NEST) were coated with anti-CD4 (GK1.5, BioLegend), followed by an incubation with 10 μL two-fold serially diluted samples to be tested. After washing, the wells were incubated with biotinylated anti-CD4 (RM4-4, BioLegend) and subsequently with HRP-coupled streptavidin. The levels of sCD4 were photometrically determined according to the manufacturer’s manual (Sino Biological). The results are presented as mean ± SEM of at least three samples.

### Quantitative real-time PCR

Total RNA was extracted with RNA extraction kit (R4012-02, Magen, CN) according to the manufacturer’s instructions. Quantitative real-time PCR(qPCR) was performed with ABI QuantStudio 1 (ThermoFisher) using SYBR RT-PCR kit (Bio-Rad). The primers used in this study were: tnf forward primer 5′-TCTTCTCCTTCCTGATCGTG and reverse primer 5′-GAAG ATGATCT GACTGCCTG; il-6 forward primer 5′-TACCCCCAGGAGAAGATT CC and reverse primer 5′-TTTTCTGCCAGTGCCTCTTT. gapdh forward primer 5′-TGCACCACCAA CTGCTTAGC and reverse primer 5′- GGCATGGACTGTGGTCATGAG. Results were normalized to *gapdh* mRNA levels.

### Immunoblot and immunoprecipitation

Where indicated, membrane proteins were extracted using Minute Plasma Membrane Protein Isolation and Cell Fractionation Kit (SM-005; Invent). Whole cell lysates were routinely prepared as previously described,^72^ and protease inhibitors cocktail (Roche) added before immunoprecipitation. Total protein concentrations measured by BCA assay (Pierce).

### Macrophage depletion, purification and adoptive transfer

To deplete macrophages, mice were *i.p*. injected with 200 μL clodronate liposomes (FormuMax, F70101C-N-10). Thioglycollate broth (*i.p*., 4% in 2 mL, 3 days, Sigma-Aldrich) was used to elicit peritoneal macrophages. To adoptively transfer macrophages, donor peritoneal macrophages (2 × 10^6^ cells, in 1 mL PBS) were *i.v*. transfused to recipient mice 48 h after liposomal ablation. Efficiencies of both depletion and reconstitution were monitored by immunofluorescent staining of liver sections with rat anti-mouse F4/80 mAb (AF647, BioLegend). Mice were then *i.p*. injected with sCD4 (10 mg/kg) 12 h after the adoptive transfer of indicated macrophages. LPS (10 mg/kg) was administered to mice 12 h after sCD4 administration.

### Duolink proximity ligation assay (PLA)

Peritoneal macrophages (PM) or BMDM cells (6 × 10^5^) were seeded on a cover slip in a 6-well plate, and serum-starved in OPTI-MEM overnight, LPS or/and sCD4 were then added at the indicated concentrations for 30 min. Cells were then fixed (4% PFA, 4 ^o^C, 15 min), quenched (0.1 M glycine in PBS, 4 ^o^C, 10 min), washed twice with cold PBS. Permeabilization step was applied for detection of cytosolic proteins (saponin solution, #P0095, Beyotime), 30 min). Cells were blocked using Doulink in situ RED starter kit (Sigma, DUO92101-1KT) for 1 h at room temperature. Primary antibodies (1 μg/mL) targeting MHCII, TLR4, SHP-2, pSHP-2) were used and inter-protein interaction was visualized with probes (DUO92101-1KT, Sigma) that binds to the primary antibodies according to the manufacturer’s protocol. Images were captured with DeltaVison microscope (60x, oil; GE), and red fluorescent dots representative of 1 inter-molecular interaction were counted (4 view fields and at least 160 cells) and analyzed with Imaris Software (Oxford Instruments). IgG isotype used as control.

### Immunofluorescence staining

Peritoneal macrophages (3 × 10^5^) were seeded on a cover slip in a 6-well plate, and serum-starved overnight in OPTI-MEM before LPS or/and sCD4 were added. Cells were then fixed, quenched and washed as PLA assays, except that 0.25% Triton X-100 was used for permeabilization, and 1% BSA in PBST for blocking. Primary antibodies against MHCII, TLR4, EEAL, LAPM1, RAB4, RCAS1, and secondary antibodies (Goat anti-Rat Alexa Fluor 647; Donkey anti-Mouse IgG Alexa Fluor 488; Goat anti-Rabbit Alexa Fluor 555) were applied for fluorescent imaging analyses (DeltaVision OMX, 60 x, oil; GE). Green fluorescent dots (10 view fields and at least 10 cells) were counted and analyzed with Imaris Software (Oxford Instruments).

### Live cell imaging of lysosomes

RAW264.7 cells (1 × 10^5^) were seeded in a glass bottom dish (35 mm) for 24 h, then transfected with GFP tagged MHC IIAβ or MHC IIAβΔCT. 24 h later, cells were spiked with lysosome tracker for 30 min. Washed and replaced with pre-warmed fresh medium, LPS (500 ng/mL) or LPS plus sCD4 (125 nM) added to cells. Live cell imaging (every 10 s for 20 min) of 4 random view fields were acquired using Olympus SpinSR10 Ixplore microscope (60x, oil). The size of the lysosome was analyzed by the Surface combined with the automatically tracking objects of interest using the provided algorithms based on Imaris Software (Oxford Instruments).

### Multiplex immunohistochemistry

In brief, 3 μm FFPE sections were deparaffinized in xylene and then rehydrated in 100%, 95%, 90%, 80%, 70% alcohol successively. Antigen unmasking was performed with a preheated epitope retrieval solution (citrate buffer, pH = 6), endogenous peroxidase was inactivated by incubation in 3% H_2_O_2_ for 20 min. Sections were then pre-incubated with 10% normal goat serum, followed by incubation of primary antibodies overnight (rabbit anti-CD4 antibody (Abcam; ab183685), rabbit anti-F4/80 antibody (CST; #70074)). Next, sections were incubated with the goat anti-rabbit HRP-conjugated secondary antibodies (Abcam; ab214880) for 60 min at room temperature. The antigenic binding sites were visualized by Opal dyes applied to each secondary antibodies. Opal-690 (FP1497001KT) and Opal-570 (FP1488001KT) were from PerkinElmer. Slices were mounted in DAPI fluoromount-G reagent (p0131, Beyotime) and imaging data were obtained with Vectra3 (20× objective, PerkinElmer). Cell-cell interactions were analyzed with Halo Software (Indica Labs).

### Statistical analysis

Statistical analysis was performed by using Log-rank (Mantel-Cox) test, Unpaired *t* test and one-way ANOVA with Dunnett’s analysis (Graphpad Prism 8). Data are presented as the mean ± SD. *P*-value < 0.05 was considered statistically significant.

## Supplementary information

Supplementary_Materials

## Data Availability

Data that support the findings of this study are available upon reasonable request to the lead contact (H.T.).
