# Supplementary material for: Soluble CD4 effectively prevents excessive TLR activation of resident macrophages in the onset of sepsis
Source: Signal Transduct Target Ther. 2023 Jun 19;8:236. doi: 10.1038/s41392-023-01438-z (PMC10277282; doi:10.1038/s41392-023-01438-z)
Supplement: Supplementary file 1 — Supplementary_Materials [file 41392_2023_1438_MOESM1_ESM.docx]

Supplementary Materials for

**Soluble CD4 effectively prevents excessive TLR activation of resident macrophages in the onset of sepsis**

Sheng-yuan Zhang^1,3,7, ♯^, Qiu-ping Xu^1, ♯^, Li-na Shi^1,7, ♯^, Shih-wen Li^1, ♯^, Wei-hong Wang^1^, Qing-qing Wang^1^, Liao-xun Lu^6^, Hui Xiao^1^, Jun-hong Wang^1^, Feng-ying Li^1^, Yin-ming Liang^6^, Si-tang Gong^5^, Hao-ran Peng^4,^*, Zheng Zhang^3,^*, Hong Tang^1,2,#,^ *

^1^ CAS Key Laboratory of Molecular Virology and Immunology, Institut Pasteur of Shanghai, Chinese Academy of Sciences, Shanghai 210031, China

^2^ State Key Laboratory for Diagnosis and Treatment of Infectious Diseases, National Clinical Research Centre for Infectious Diseases, Collaborative Innovation Centre for Diagnosis and Treatment of Infectious Diseases, The First Affiliated Hospital, Zhejiang University School of Medicine, Hangzhou 310003, China

^3^ The Third People’s Hospital of Shenzhen, Shenzhen 518112, China

^4^ Department of microbiology, Naval Medical University, Shanghai 200433, China,

^5^ The Joint Center of Translational Medicine, Guangzhou Women and Children’s Medical Center and Institut Pasteur of Shanghai, Guangzhou 510623, China

^6^ The Laboratory of Genetic Regulators in The Immune System, Xin-xiang Medical University, Henan Province 453003, China

^7^ University of Chinese Academy of Sciences, Beijing 100101, China

^♯^ equal contributions

*Address all correspondence to H.P (phran@126.com), Z.Z (zhangzheng1975@aliyun.com) or H.T (h_tang@zju.edu.cn).

^#^ Lead contact

**This PDF file includes:**

Figures. S1 to S9

Tables S1 to S5

Figure. S1.


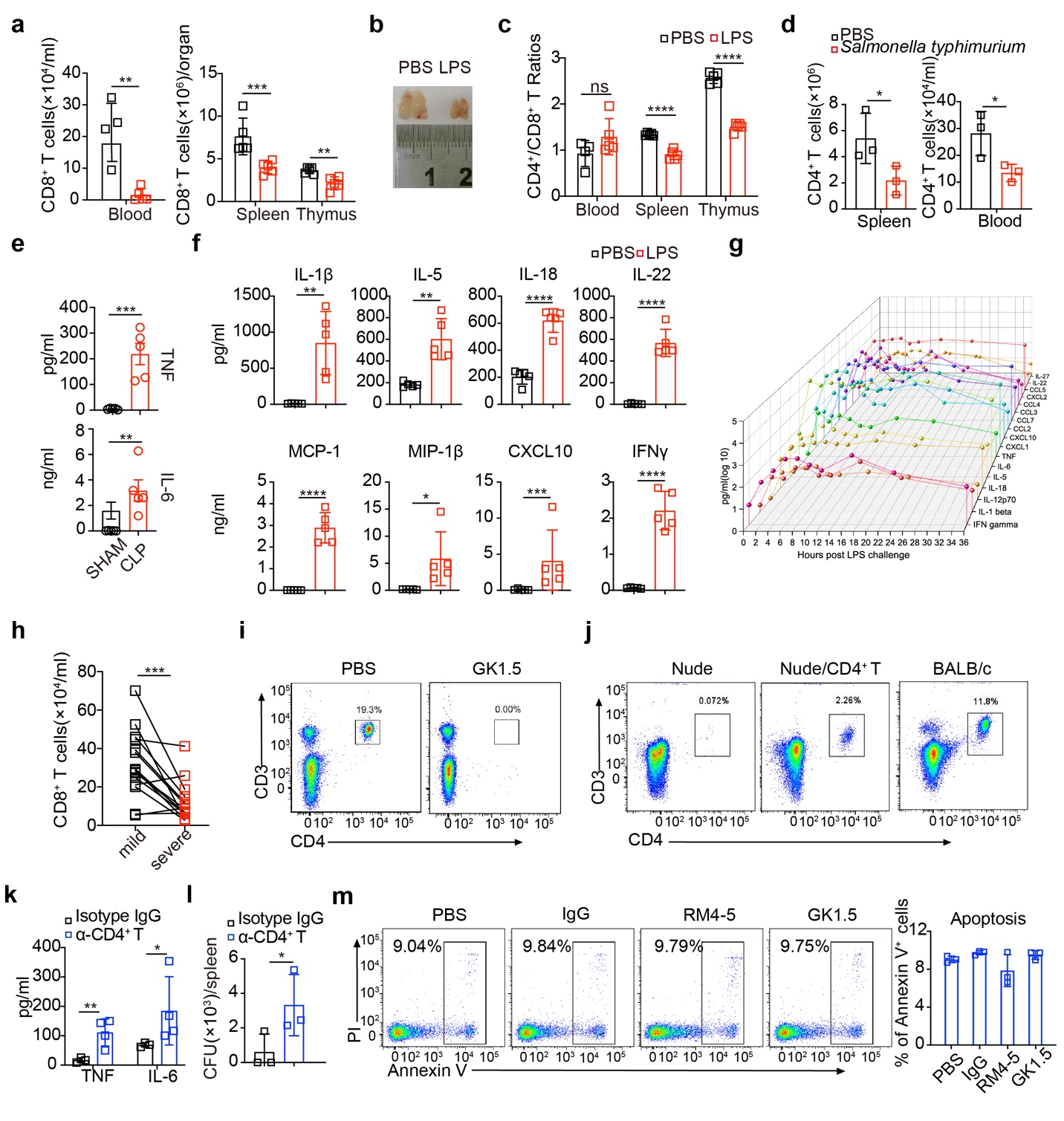


**Figure. S1. CD4^+^ T lymphopenia in Sepsis Associates with Impaired Immune Response.** Related to Figure 1 and Figure 2.

Twelve hours after WT mice (dots) were *i.p.* injected with LPS or PBS as sham, (**a**) absolute numbers of CD8^+^ T cells and (**c**)the ratios of CD4^+^ / CD8^+^ T in the indicated organs analyzed by FACS and (**b**) representative of the size of the thymus. (**d**) Absolute numbers of CD4^+^ T cells in the spleen or blood were measured post 60h of *Salmonella typhimurium* infection. (**e**) Serum TNF and IL-6 were measured 12 h after CLP. Serum PICC measured by Luminex at 12h (**f**) or indicated time (**g**) post LPS challenge by *i.p.*. (**h**) Absolute numbers of peripheral CD8^+^ T cells of COVID-19 patients (n = 15). Flow cytometric analyses of *peripheral* CD3^+^CD4^+^ T cells (**i**) 3 days after GK1.5 antibody ablation, or (**j**) 7 days after nude mice were adoptively transferred with CD4^+^ T cells (2 x 10^6^ cells/mouse). Balb/c WT mice as control. WT mice were pre-treated with GK1.5 or isotype control antibody (each dot) 2 days before *Salmonella typhimurium* oral gavage. 60 h after infection, (**k**) serum cytokines and (**l**) bacterial loads in the spleen were measured. (**m**) Annexin V and PI staining of CD4^+^ T cells 18 h after co-cultured with the indicated CD4 antibodies or IgG2a isotype. Mean ± SD are shown; n = 3-5 mice used where indicated; Unpaired t test. *ns,* > 0.05; *, *P* < 0.05; **, *P* < 0.01; ***, *P* < 0.001; ****, *P* < 0.0001).

Figure. S2.


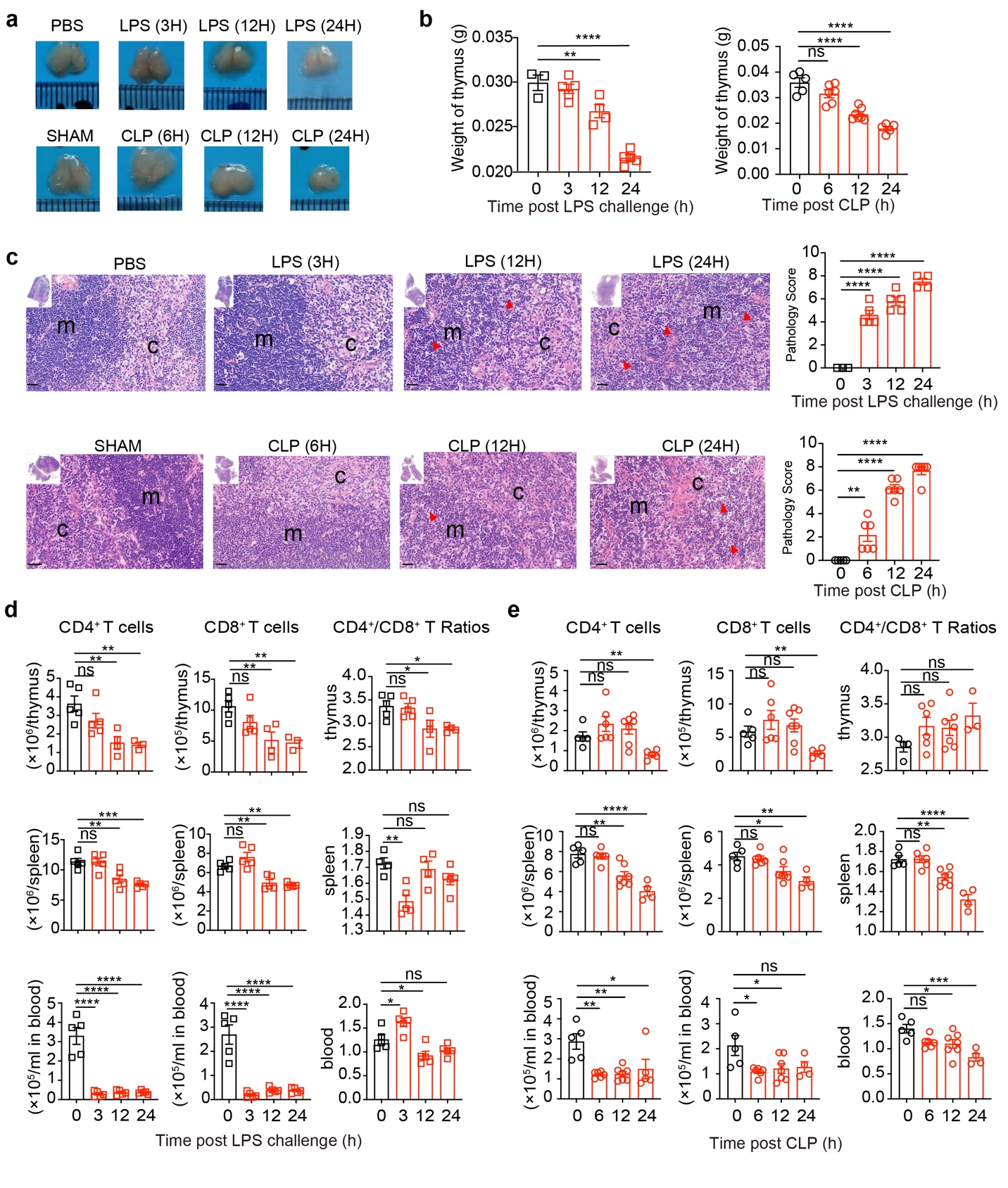


**Figure. S2. CD4^+^ and CD8^+^ T lymphopenia in Sepsis Associates with thymus injury.** Related to Figure 1

(**a-b**) Representative of the size and weight of the thymus at the indicated time post LPS or CLP challenge. (**c**) Representative sections and pathology score of thymus (with respective magnifications of areas of interest) at indicated time post LPS or CLP challenge. Red arrow shows the cell death area; m (medulla); c (cortex). Bar = 50 μm. (**d-e**) CD4^+^, CD8^+^ T cell counts and the ratios of CD4^+^ / CD8^+^ T in the indicated organs analyzed by FACS at indicated time post LPS (**d**) or CLP (**e**) challenge. Mean ± SD are shown; n = 3-6 mice used where indicated; Statistics (ns, *P* > 0.05; *, *P* < 0.05; **, *P <* 0.01; ***, *P* < 0.001; ****, *P* < 0.0001): one-way ANOVA with Dunnett’s analysis.

Figure. S3.


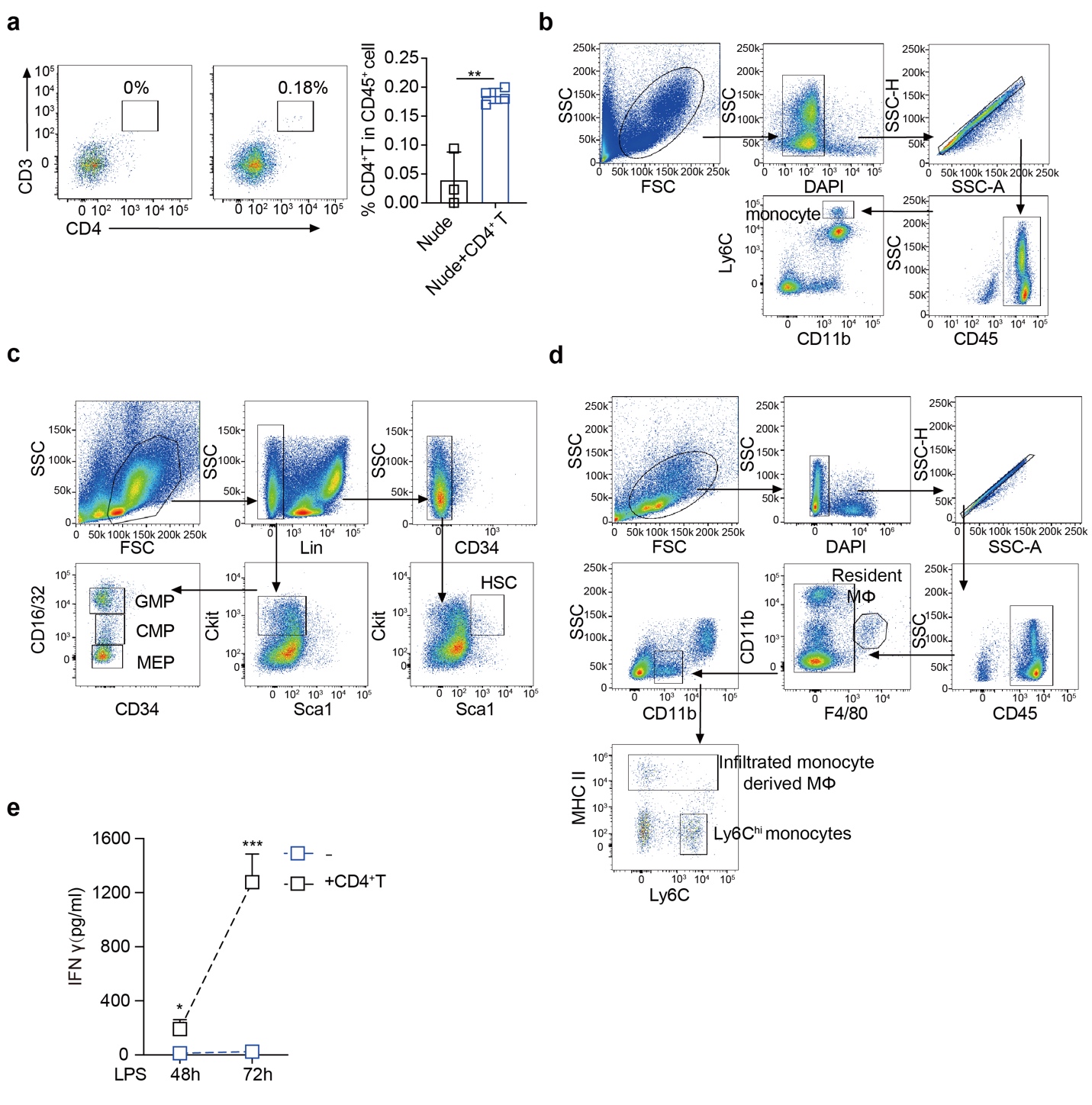


**Figure. S3. CD4^+^ T cells in myelopoiesis and mono-macrophages gating strategies.** Related to Figure 2.

(**a**) Efficiency of CD4^+^ T cell reconstitution in bone marrow. 7 days after nude mice (each dot) were i.p. transferred with naïve CD4^+^ T cells (2 x 10^6^ each mouse), the number of CD4^+^ T cells reconstituted in bone marrow was measured by flow cytometry. Gating strategies of (**b**) Ly6C^hi^ monocytes in BM/blood/spleen/liver, (**c**) hematopoietic stem cells and progenitor cells in BM. HSC (lin-CD34^low^Sca-1^+^c-kit^+^), GMP (CD34^hi^CD16/32^hi^), CMP (CD34^high^CD16/32^low^) and MEP (CD34^low^CD16/32^low^). Lineage cocktail (lin) included anti-CD3e, anti-CD11b, anti-B220 and anti-TER119. (**d**) Gating strategy of macrophages in the spleen and liver, resident macrophages (CD11bl^ow^ F4/80^hi^), infiltrated macrophages (CD11b^int^ F4/80- MHC II^+^). (**e**) Luminex measurement of IFNγ in the supernatants after BM-derived monocytes and CD4^+^ T cells were co-cultured for the indicated time, in the presence of LPS. Mean ± SD are shown; n = 3-4 mice used where indicated; Unpaired t test. ns, *P* > 0.05; *, *P* < 0.05; **, *P* < 0.01; ***, *P* < 0.001.

Figure. S4.


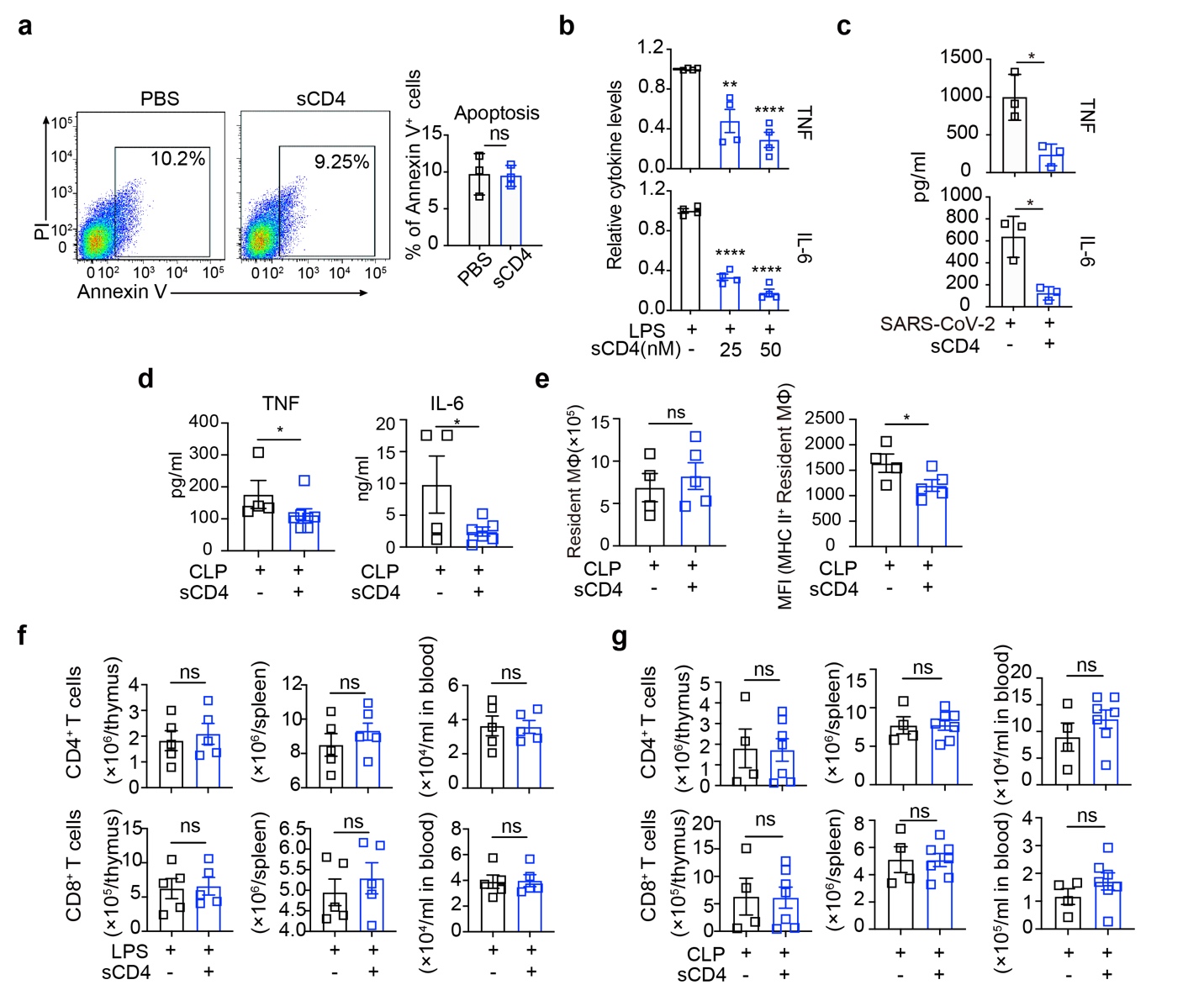


**Figure. S4. sCD4 suppressed TLR4 activation in both SARS-COV-2 infection and CLP modles.** Related to Figure 3.

(**a**) sCD4 ligation of MHC II did not cause macrophage cell death. Annexin V/PI staining of macrophages 18 h after co-cultured with sCD4 and LPS. (**b**) Measurement of TNF and IL-6 after BMDC cells (1 x 10^5^) were stimulated with LPS (100 ng/mL) in the presence of sCD4 for 16 h. (**c**) TNF and IL-6 in supernatants 24 h after PBMC were infected with SARS-CoV-2 (MOI = 0.1) in the presence of sCD4. (**d**) TNF and IL-6 in supernatants, (**e**) resident macrophages, and activated macrophages (MHC II^+^) in spleen were analyzed 12h after CLP. Absolute numbers of CD4^+^ and CD8^+^ T cells in the indicated organs of mice pre-treated with sCD4 or PBS challenged by LPS *i.p.* for 12h (**f**) or by CLP for 12h (**g**). Mean ± SD are shown; n = 3-7 mice used where indicated; Unpaired t test. ns, *P* > 0.05; *, *P* < 0.05; **, *P* < 0.01; ****, *P* < 0.0001.

Figure. S5.


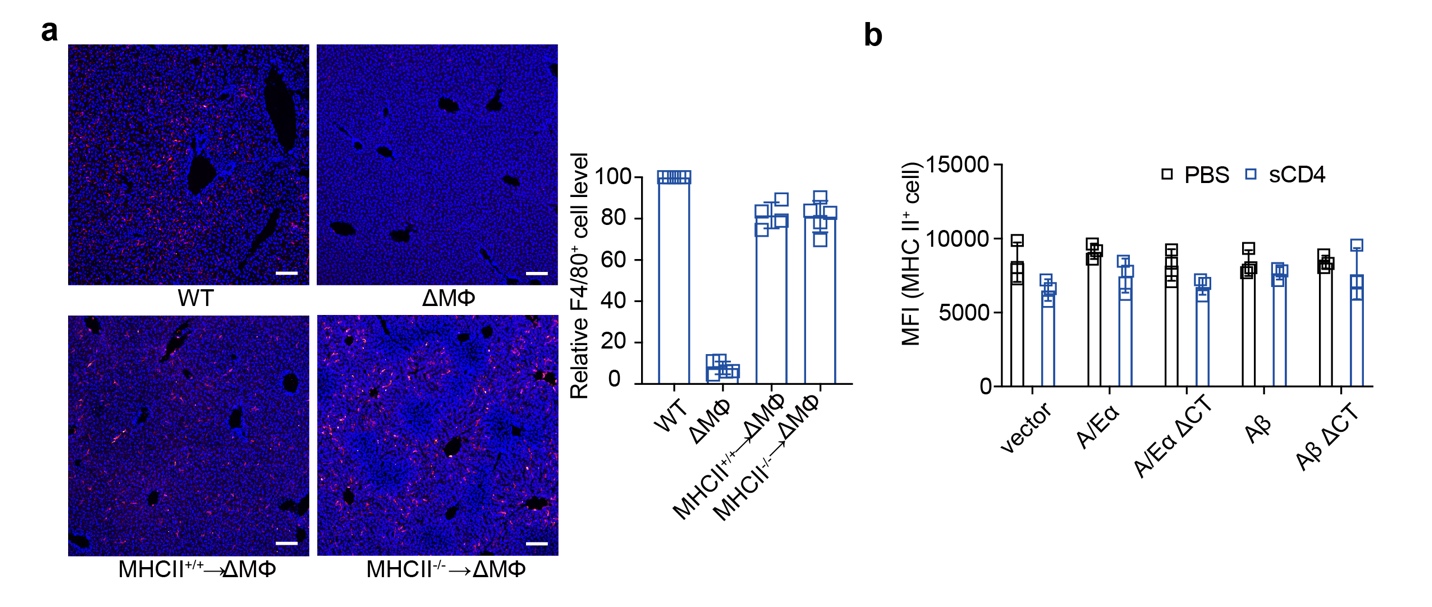


**Figure. S5. Macrophage reconstitution efficiencies in the liver.** Related to Figure 4**.**

Macrophages depleted mice (ΔMΦ) were i.v. infused with MHCII+/+ or MHCII –/– peritoneal macrophages (2 × 10^6^). Total macrophages numbers reconstituted in the liver were measured 24 h post infusion by (**a**) immunofluorescence staining of frozen sections using F4/80 antibody (red). The nuclei counter stained with DAPI (blue). Bar = 100 μm. Reconstitution efficiencies shown as the percentage of F4/80+ macrophages compared to those in naïve WT liver. (**b**) FACS analysis of GFP-tagged MHC II subunits or mutants (ΔCT) 24 h after transiently transfected in peritoneal macrophages. MFI of GFP after stimulation of LPS or sCD4 plus LPS were compared. CT, cytoplasmic tails. Mean ± SD are shown; n = 3-4 mice used where indicated.

Figure. S6.


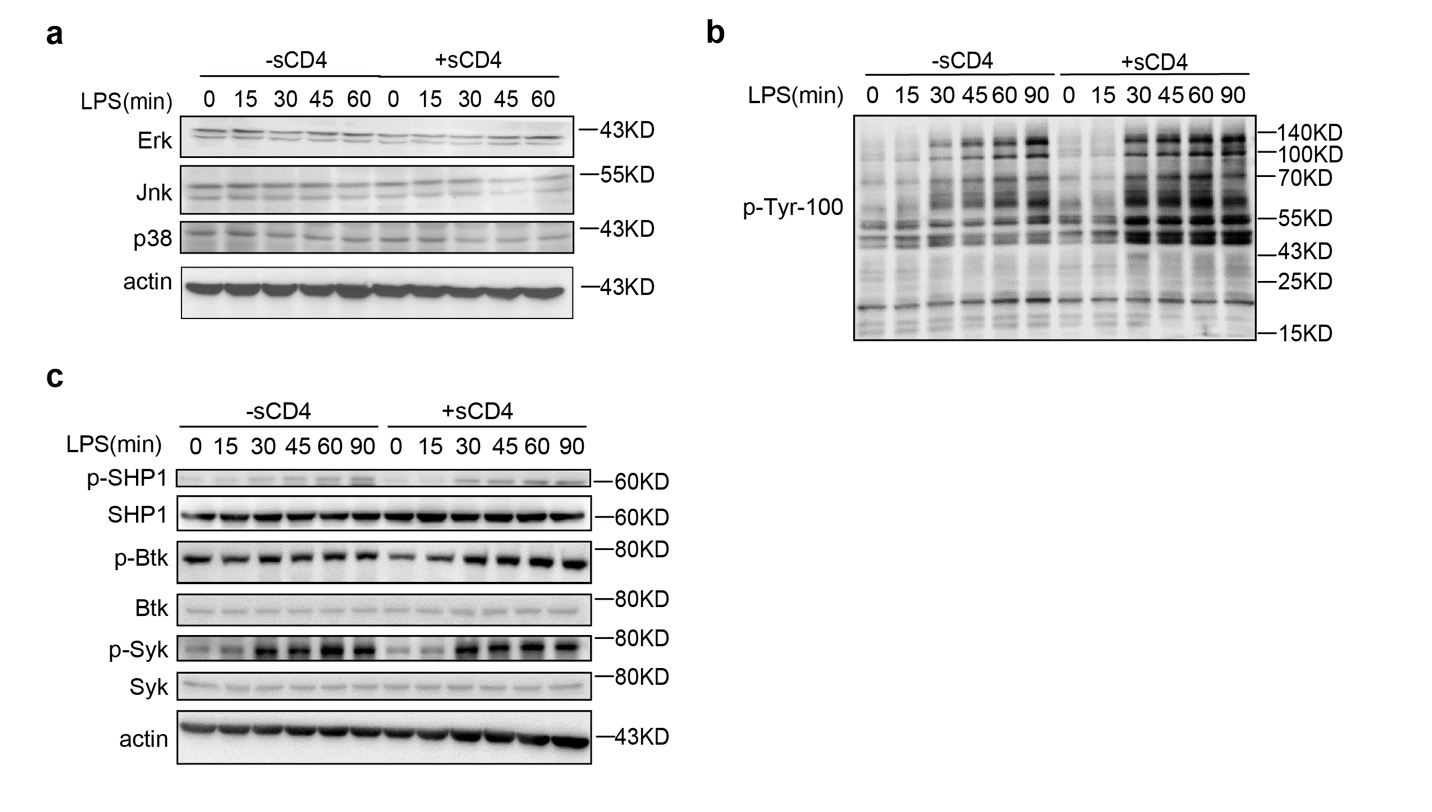


**Figure. S6. sCD4 dampened TLR3 and TLR4 pro-inflammatory signaling.** Related to Figure 5.

(**a**) Protein loadings of the indicated kinases in BMDM were measured by Western blotting. (**b**) Pre-incubation of sCD4 with BMDM elevated the overall protein tyrosine phosphorylation by LPS stimulation. Pan phospho-Tyrosine antibody (p-Tyr-100) was used, and 30 μg total protein lysates loaded in each lane. (**c**) Western bloting of activation of indicated protein tyrosine phosphatases, using phospho- and total-phosphatase specific antibodies.

Figure. S7.


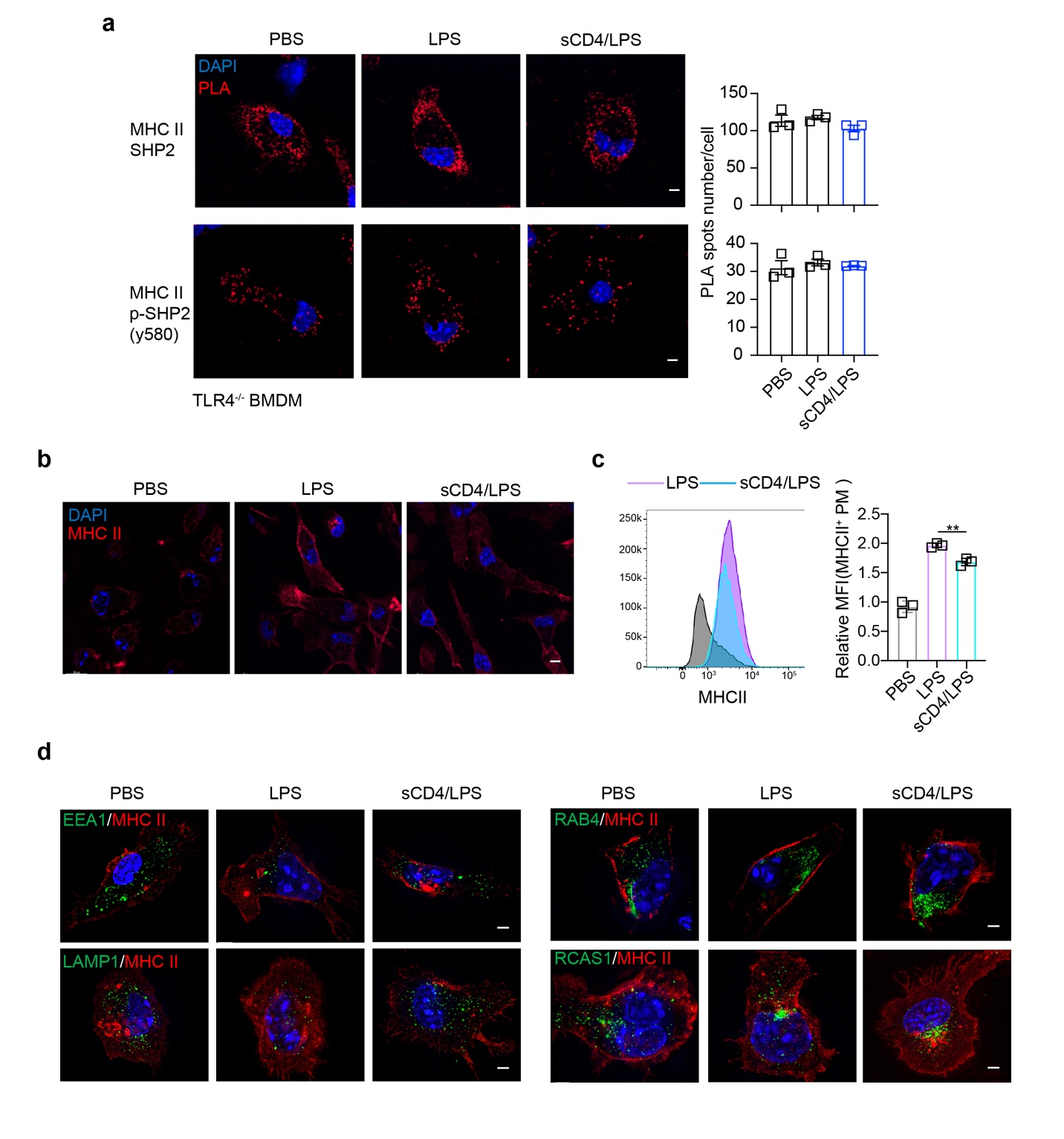


**Figure. S7. MHC II expression and trafficking in macrophages was modulated by sCD4.** Related to Figure 6.

(**a**) MHC II recruitment of SHP2 was TLR4 dependent. Duolink quantification of the indicted protein-protein association (red dots) after TLR4^-/-^ BMDM were treated with LPS or sCD4 plus LPS. Bar = 5 μm. (**b**) FACS analysis of surface MHC II expression in peritoneal macrophages 12 h after mice were *i.p.* injected with LPS (100 ng/mL) or LPS plus sCD4 (25 nM). Fluorescent microscopic analysis of (**c**) surface MHC II (red, Bar = 10 μm) and (**d**) colocalization of indicated organelles (green) with MHCII (red, Bar = 5 μm), after peritoneal macrophages were treated with LPS (100 ng/mL) in the absence or presence of sCD4 (25 nM) in plates. Mean ± SD are shown; n = 3 mice used where indicated; Statistics (ns, *P* > 0.05; *, *P* < 0.05): Unpaired t test.

Figure. S8.


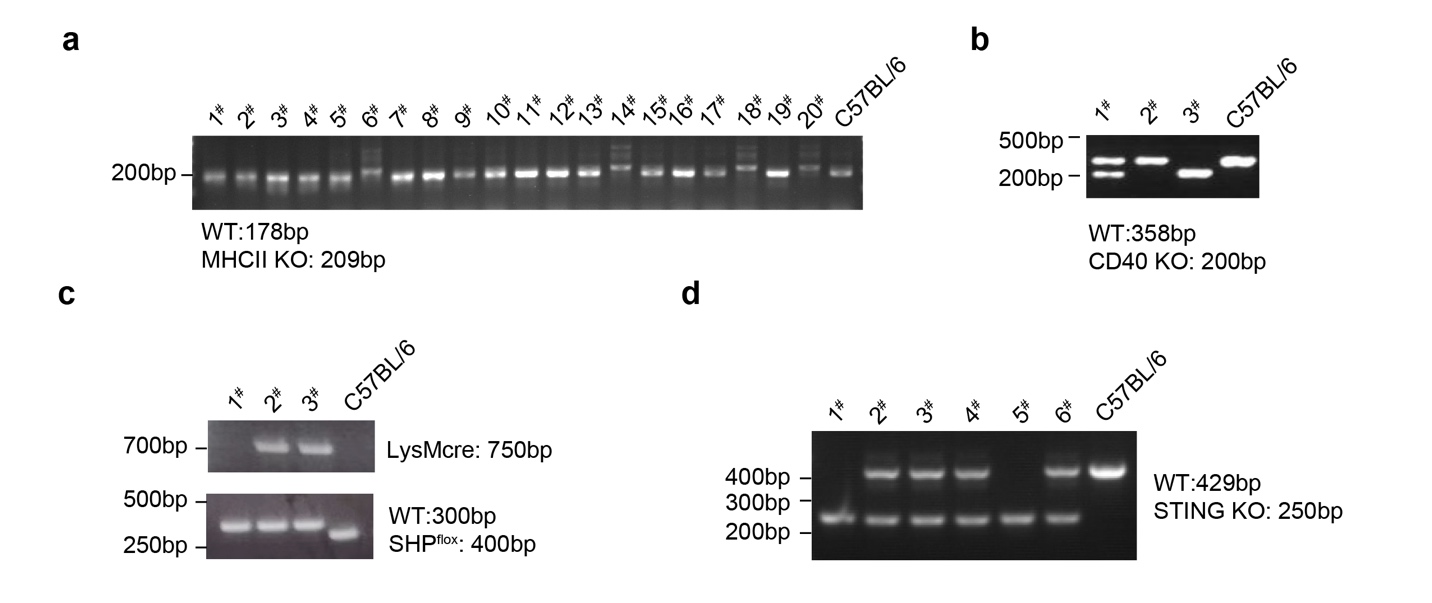


**Figure. S8. PCR genotyping of indicated gene knockout mice relevant to this study.** Related to Figure 4 and Figure 5.

PCR genotyping of MHC II KO (**a**), CD40 KO (**b**), SHP2^flox^×LysMzcre (**c**), and STING KO (**d**) mice. Genomic DNA from tail clips was used as template for primers specific PCR. DNA sample from C57BL/6 served as controls. Samples were electrophoresed through a 2% agarose gel and then imaged.

Figure. S9.


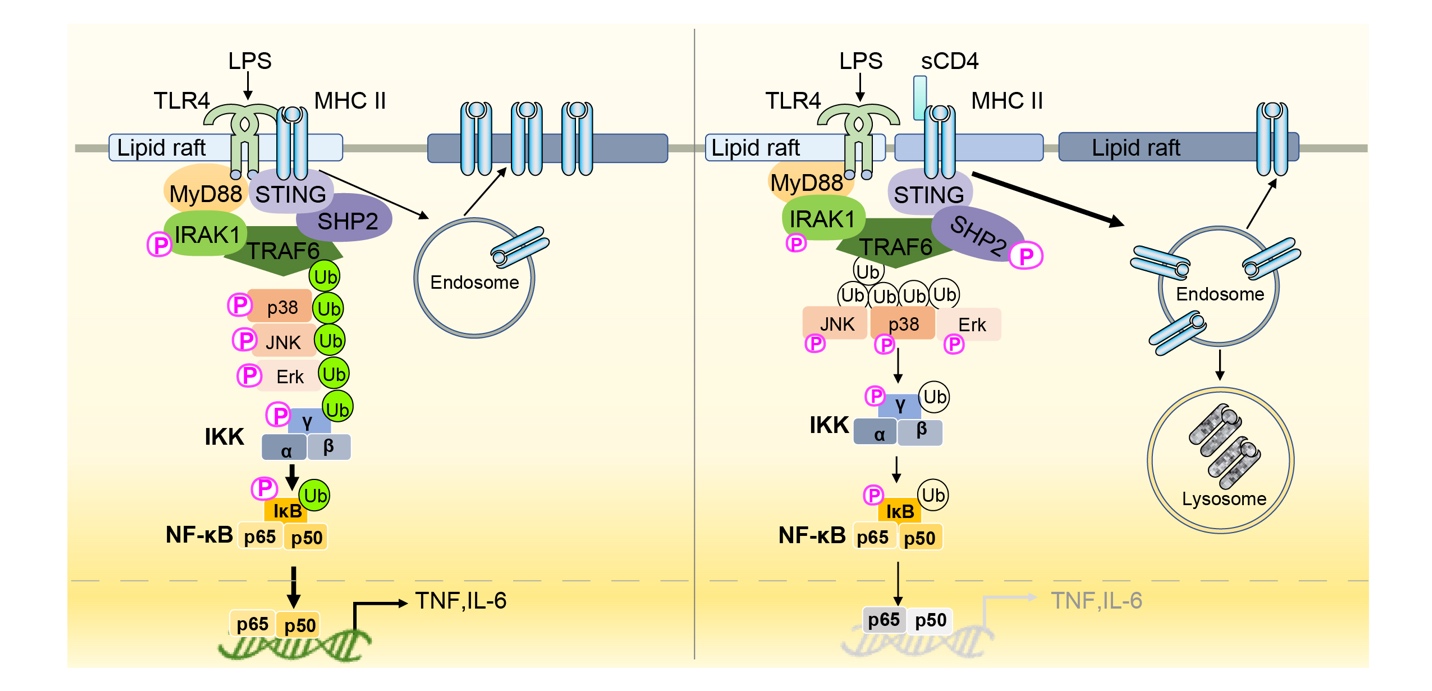


**Figure. S9.** **Summary diagram of signaling pathway of this study: the pro-inflammatory LPS/TLR4 raft compartments are disrupted by sCD4 engagement of MHCII membrane domains.**

LPS is sensed by TLR4/MyD88, which recruits and phosphorylates IRAK1 to activate MAPK and NF-κB signaling cascade in septic inflammation. It is known previously that STING may associate with the membrane proximal/cytoplasmic domain of MHC II in APC cells, and SHP2 regulates TLR/MyD88 signaling by contact with TRAF6, we find that TLR4/MHCII pro-inflammatory raft compartments are disrupted by sCD4 engagement of MHC II. Formation of sCD4/MHCII membrane domains activate SHP2 and elevate MHCII/SHP2/TRAF6 interaction to specifically inhibit phosphorylation of IRAK1, as well as Erk, Jnk, p38 and IκBα, to prevent macrophages from over-activated by LPS. sCD4 engagement also promoted endosome-lysosome trafficking of MHCII, SHP2 and STING, to downregulate LPS/TLR4 inflammation.

Table S1. Clinical data of the enrolled patients

Table S2. sgRNA sequences used to target CD40 gene

| **Name** | **gRNA sequences** |
| --- | --- |
| sgRNA-1 | 5’-CTTCTGATCTCGCCCTGCGATGG-3’ |
| sgRNA-2 | 5’-GCCTCGGCTGTGCGCGCTATGGG-3’ |
| sgRNA-3 | 5’-TGCTTGTTGACAGCGGTGAGTGG-3’ |
| sgRNA-4 | 5’-TCAAGGGCTTCGGGTTAAGAAGG-3’ |
| sgRNA-5 | 5’-CTGCACCAGCAAGGATTGCGAGG-3’ |

Table S3. A series of primers for mice genotyping

| **Name** | **sequences** |
| --- | --- |
| CD40-forword | 5’- CAGATGACGTGAGGAGTGGG -3’ |
| CD40-reverse | 5’- GAGCCCAGGTCCAAATCCAA-3’ |
| MHC II-forword-1 | 5’- CGGAAGTGCTTGACATTGG -3’ |
| MHC II-reverse-1 | 5’- GTATTGACCGATTCCTTGCG -3’ |
| MHC II-forword-2 | 5’- AACCTTCAGGATCTGTGATCC -3’ |
| MHC II-reverse-2 | 5’- GTGGCTGTTGCCTTAAGACC -3’ |
| SHP2^flox^-forword | 5’- ACGTCATGATCCGCTGTCAG -3’ |
| SHP2^flox^-reverse | 5’- ATGGGAGGGACAGTGCAGTG -3’ |
| LysMcre-forword | 5’- CCCAGAAATGCCAGATTACG -3’ |
| LysMcre -reverse | 5’- CTTGGGCTGCCAGAATTTCTC -3’ |
| STING-forword-1 | 5’- CTCCTAGACAGGTGCTGTAGGATG -3’ |
| STING- forword-2 | 5’- TGGAGACCACAGAGGGTTACCTG -3’ |
| STING- reverse | 5’- AAGGGTTATTGAATATGATCGGA -3’ |

Table S4. Histopathological Scoring of Lung and Thymus injury

| **Histopathological Scoring of Lung injury** | | | | | |
| --- | --- | --- | --- | --- | --- |
| **Score** | **Thickness of alveolar** | **Infiltration of neutrophils** | | **Alveolar congestion** | |
| 0 | Absent | Absent | | Absent | |
| 1 | Discrete | Discrete | | Small foci | |
| 2 | Moderate | Moderate | | Large foci | |
| 3 | Severe | Severe | | Diffuse | |
| **Histopathological Scoring of THYMUS** | | | | | |
| **Score** | **Cell density** | **Cell death** | **The ratio of cortex to medulla** | | **corticomedullary (CM) junction** |
| 0 | High | Absent | Normal | | Distinct |
| 1 | Medium | Moderate | / | | Blurry |
| 2 | Low | Severe | Abnormal | | Diffuse |

Table S5. Antibodies used in FACS assay

| **Antibodies** | **Source** | **Identifier** |
| --- | --- | --- |
| FITC anti-mouse CD3ε | BioLegend | 100305 |
| APC anti-mouse CD4 | BioLegend | 100515 |
| PE anti-mouse CD8a | BioLegend | 100707 |
| APC anti-mouse CD64 | BioLegend | 139306 |
| FITC anti-mouse Ly-6C | BioLegend | 128006 |
| PerCP/Cyanine5.5 anti-mouse/human CD11b | BioLegend | 101227 |
| Brilliant Violet 510™ anti-mouse CD45 | BioLegend | 103138 |
| PerCP/Cyanine5.5 anti-mouse CD86 | BioLegend | 105028 |
| APC/Cyanine7 anti-mouse CD45 | BioLegend | 103116 |
| PE anti-mouse I-A/I-E | BioLegend | 107607 |
| Brilliant Violet 605™ anti-mouse CD11c | BioLegend | 117334 |
| Brilliant Violet 510™ anti-mouse/human CD11b | BioLegend | 101263 |
| APC anti-mouse F4/80 | BioLegend | 123115 |
| Alexa Fluor® 700 anti-mouse F4/80 | BioLegend | 123129 |
| PE/Cyanine7 anti-mouse/human CD11b | BioLegend | 101215 |
| TruStain FcX™ PLUS (anti-mouse CD16/32) | BioLegend | 156603 |
| PE/Cyanine7 anti-mouse CD16/32 | BioLegend | 101329 |
| FITC anti-mouse CD117 (c-kit) | BioLegend | 135115 |
| APC anti-mouse CD34 | eBioscience | 11-0341 |
